# Supplementary material for: Cucurbit[8]uril-based water-dispersible assemblies with enhanced optoacoustic performance for multispectral optoacoustic imaging
Source: Nat Commun. 2023 Jul 3;14:3918. doi: 10.1038/s41467-023-39610-2 (PMC10317952; doi:10.1038/s41467-023-39610-2)
Supplement: Supplementary file 1 — Supplementary Information [file 41467_2023_39610_MOESM1_ESM.pdf]

Supplementary Information

Cucurbit[8]uril-Based Water-Dispersible Assemblies with Enhanced Optoacoustic Performance for Multispectral Optoacoustic Imaging

Wu et al.

Supplementary Methods

Materials. Chondroitin sulfate A sodium salt from bovine trachea, cucurbit[n]uril hydrate (n = 6, 7, or 8), cyclohexanone, 2-hydroxybenzaldehyde, iodoethane, iodomethane, 2-methylbenzothiazole, 4-methylpyridine, piperidine, tetrabutylammonium chloride, and anhydrous solvents such as acetic anhydride (Ac_2O), acetonitrile (MeCN), 1-butanol (BuOH), chloroform ($CHCl_3$), 1,2-dichlorobenzene (*o*-DCB), N,N-dimethylformamide (DMF), and ethanol (EtOH) were purchased from Sigma-Aldrich. The solvents including acetone, dichloromethane (DCM), diethyl ether, ethyl acetate (EtOAc), and methanol (MeOH) were of analytical grade. Cesium carbonate (Cs_2CO_3), phosphorus tribromide, and trifluoroacetic acid (TFA) were procured from Tokyo Chemical Industry Co. Ltd. 2,5-Dihydroxy-1,4-benzenedicarboxaldehyde was obtained from BLD Pharmatech Ltd. and used without further purification. PEG_{5000}-functionalized gold nanorods (40 × 100 nm) were purchased from Nanjing Nanoeast Biological Technology Co. Ltd. Triple-distilled water was used throughout the experiments.

Instruments. ^1H and ^{13}C NMR spectra and 2D NOESY were measured with a Bruker BBFO 400 Spectrometer. DQF-COSY, HSQC and DOSY spectra were measured on a JEOL ECZL500 Spectrometer. The electronic spray ionization (ESI) mass spectra were recorded on a ThermoFinnigan LCQ quadrupole ion trap mass spectrometer. MALDI TOF mass spectra was recorded on an AB Sciex MALDI-TOF/TOF 5800 system. The absorption spectra were collected on UV-3600 Shimadzu UV-Vis-NIR Spectrophotometer and Hitachi U-3010 UV-vis spectrophotometer. Transmission electron microscopy (TEM) images were collected on JEOL JEM-1400. The size distributions and zeta potential of nanoagents were determined through dynamic light scattering (DLS) by using Malvern Instruments Zetasizer Nano-S at 25 °C. MTT assay was measured with a Tecan's Infinite M200 microplate reader. Flow cytometry was taken on a BD FortessaX20 5-laser cytometer. Confocal laser scanning microscopy (CLSM) images were acquired by ZEN 2 (blue edition) software of Carl Zeiss LSM 800. Optoacoustic imaging was performed on an inVision128 multispectral optoacoustic tomographic (MSOT) imaging system (iThera Medical GmbH) and fluorescence imaging was conducted on an IVIS spectrum

imaging system.

Synthesis of Compound 1. 4-Methylpyridine (3.0 mL, 30 mmol) and iodomethane (2.4 mL, 30 mmol) were dissolved in anhydrous acetonitrile (10 mL) in a sealed tube. After being refluxed at 90 °C overnight, the mixture was poured into cold diethyl ether (200 mL). Then, the suspension was filtered, and the precipitate was washed with a small amount of ethyl acetate three times. The product was collected and dried in vacuum as a red solid (3.22 g, yield: 88%). ^{1}H NMR (400 MHz, $CDCl_3$): δ 9.27-9.26 (d, 2H), 7.90-7.88 (d, 2H), 4.94-4.89 (q, 2H), 2.66 (s, 3H), 1.70-1.67 (t, 3H). ESI-MS (m/z) 121.95 $[M]^+$.

Synthesis of Compound 2. 2-Methylbenzothiazole (1.22 mL, 10 mmol) and iodomethane (1.85 mL, 30 mmol) were dissolved in anhydrous acetonitrile (10 mL) in a sealed tube. After being refluxed at 90 °C overnight, the mixture was poured into cold diethyl ether (200 mL). Then, the suspension was filtered, and the precipitate was washed with a small amount of ethyl acetate three times. The product was collected and dried in vacuum as a pale blue solid (1.51 g, yield: 92%). ^{1}H NMR (400 MHz, DMSO-d_6): δ 8.45-8.43 (d, 1H), 8.30-8.28 (d, 1H), 7.92-7.88 (t, 1H), 7.82-7.79 (t, 1H), 4.20 (s, 3H), 3.17 (s, 3H). ESI-MS (m/z) 163.92 $[M]^+$.

Synthesis of Compound 3. To a mixture of anhydrous chloroform (50 mL) and N,N-dimethylformamide (11.2 mL, 144.6 mmol), phosphorus tribromide (12.4 mL, 130.5 mmol) was added dropwise at 0 °C within 1 h. Then, the mixture was warmed up to room temperature and stirred for another 1 h. After that, cyclohexanone (5 mL, 48.3 mmol) was added into the mixture via a syringe, and the resulting solution was stirred at room temperature for additional 16 h. After the reaction was completed, the mixture was poured onto ice water (200 mL), neutralized with solid sodium bicarbonate, and extracted with dichloromethane 3-5 times. The combined organic layers were dried over anhydrous sodium sulfate, filtered and concentrated in vacuo to provide an orange oil, Compound 3 (7.54 g, 83 %), which was used directly for further reactions without any characterization due to its instability.

Synthesis of Compound 4. Compound 3 (1.84 g, 9.8 mmol) and 2-hydroxybenzaldehyde (0.8

g, 6.5 mmol) were dissolved with anhydrous DMF (20 mL) in a two-neck round-bottom flask. After adding cesium carbonate (2.1 g 6.5 mmol) into the mixture, the flask was purged with dry argon gas three times. Then the medium was stirred at room temperature for 24 h and a distinct yellow spot appeared on the TLC plate (hexane: acetone = 10:1). The reaction mixture was filtered, and the filtrate was concentrated in vacuo. The resulting residue was redissolved with dichloromethane (50 mL) and washed with H_2O (20 mL) three times. The organic layer was dried over anhydrous sodium sulfate, filtered and concentrated in vacuo. The crude product was purified in a silica-gel column using hexane/acetone (10:1 v/v) as eluent to give the product Compound 4 as a yellow solid (1.20 g, yield: 87%). ^{1}H NMR (400 MHz, DMSO-d_6): δ 10.25 (s, 1H), 7.39-7.34 (m, 2H), 7.23-7.21 (m, 1H), 7.17-7.12 (m, 1H), 6.99 (s, 1H), 2.60-2.56 (m, 2H), 2.31-2.27 (m, 2H), 1.66-1.59 (m, 2H). ESI-MS (m/z) 213.06 $[M+H]^+$.

Synthesis of Compound 5. Compound 3 (5.64 g, 30 mmol) and 2,5-dihydroxy-1,4-benzenedicarboxaldehyde (1.66 g, 10 mmol) were dissolved with anhydrous DMF (50 mL) in a two-neck round-bottom flask. After adding cesium carbonate (6.52 g 20 mmol) into the mixture, the flask was purged with dry argon gas three times. Then, the medium was stirred at room temperature for 72 h. The reaction mixture was concentrated to around 20 mL in vacuo and then poured into water (200 mL). After vigorous sonication for 10 min, the suspension was filtered. The precipitate was then washed with methanol, acetone and dichloromethane successively. The product was collected and dried in vacuum as a reddish orange solid (2.91 g, yield: 84%). ^{1}H NMR (400 MHz, $CDCl_3$): δ 10.34 (s, 2H), 6.89 (s, 2H), 6.64 (s, 2H), 2.63-2.60 (t, 4H), 2.47-2.44 (t, 4H), 1.77-1.71 (m, 4H). MALDI TOF-MS (m/z) 347.1 $[M+H]^+$.

Synthesis of Compound 6 (XP). Compound 4 (106 mg, 0.5 mmol) and Compound 1 (125 mg, 0.5 mmol) were dissolved with anhydrous ethanol (5 mL) in a sealed tube. After the addition of piperidine (20 μL), the solution was refluxed at 85 °C for 12 h. After cooling to room temperature, the solvent was evaporated in vacuum and the crude product was purified in a silica-gel column using dichloromethane/methanol (15:1 v/v) as eluent. In order to be accurately weighed for the interaction with the host molecules, the purified product was further anion-exchanged by using tetrabutylammonium chloride to afford Compound 6. The product

Compound 6 (XP) was finally obtained as a dark color solid (114 mg, yield: 72%). ^{1}H NMR (400 MHz, CD_3OD): δ 8.57-8.55 (d, 2H), 8.32-8.28 (d, 1H), 7.97-7.95 (d, 2H), 7.31-7.27 (m, 1H), 7.24-7.22 (m, 1H), 7.20-7.18 (m, 1H), 7.09-7.05 (m, 1H), 6.67 (s, 1H), 6.60-6.57 (d, 1H), 4.49-4.43 (q, 2H), 2.62-2.59 (t, 2H), 2.57-2.54 (t, 2H), 1.87-1.81 (m, 2H), 1.62-1.58 (t, 3H). ^{13}C NMR (100 MHz, CD_3OD): δ 156.01, 154.51, 154.09, 143.93, 137.41, 131.67, 130.84, 127.74, 126.20, 124.84, 123.93, 123.24, 119.34, 116.26, 112.82, 56.49, 30.70, 25.56, 21.88, 16.63. ESI-MS (m/z) 316.15 $[M]^+$.

Synthesis of Compound 7 (XBTZ). Compound 4 (106 mg, 0.5 mmol) and Compound 2 (146 mg, 0.5 mmol) were dissolved with anhydrous acetic anhydride (5 mL) in a sealed tube. After the addition of piperidine (20 μL), the solution was stirred at 80 °C for 12 h. After cooling to room temperature, the solvent was evaporated in vacuum and the crude product was purified in a silica-gel column using dichloromethane/methanol (20:1 v/v) as eluent. In order to be accurately weighed for the interaction with the host molecules, the purified product was further anion-exchanged by using tetrabutylammonium chloride to afford Compound 7. The product Compound 7 (XBTZ) was finally obtained as a dark purple solid (109 mg, yield: 61%). ^{1}H NMR (400 MHz, DMSO-d_6): δ 8.35-8.31 (d, 1H), 8.28-8.26 (d, 1H), 8.11-8.09 (d, 1H), 7.80-7.76 (t, 1H), 7.69-7.66 (t, 1H), 7.49-7.44 (m, 2H), 7.37-7.35 (d, 1H), 7.25-7.20 (m, 2H), 7.01-6.98 (d, 1H), 4.18 (s, 3H), 2.69-2.64 (m, 4H), 1.82 (m, 2H). ^{13}C NMR (100 MHz, DMSO-d_6): δ 170.59, 156.26, 152.01, 142.07, 141.86, 130.80, 129.90, 128.94, 128.80, 127.47, 127.26, 126.82, 124.68, 123.71 121.45, 115.94, 115.33, 112.33, 107.44, 35.36, 28.75, 24.11, 19.90. ESI-MS (m/z) 358.16 $[M]^+$.

Synthesis of compound 8 (DXP). Compound 5 (86.5 mg, 0.25 mmol) and Compound 1 (156 mg, 0.625 mmol) were added into a tube. Then, anhydrous 1,2-dichlorobenzene (5 mL), 1-butanol (3 mL) and piperidine (20 μL) were added into the tube. After sonication for 5 min, the tube was sealed and the mixture was stirred at 110 °C for 12 h. After cooling to room temperature, the solvent was evaporated in vacuum and the crude product was purified in a silica-gel column using dichloromethane/methanol (20:1 v/v) with 1% TFA as eluent. In order to be accurately weighed for the interaction with the host molecules, the purified product was

further anion-exchanged by using tetrabutylammonium chloride to afford Compound 8. The product Compound 8 (DXP) was finally obtained as a dark blue solid. (36 mg, yield: 26%). ^{1}H NMR (400 MHz, CD_3OD): δ 8.58-8.57 (d, 4H), 8.27-8.23 (d, 2H), 7.97-7.95 (d, 4H), 7.02 (s, 2H), 6.65 (m, 2H), 6.63-6.59 (m, 2H), 4.49-4.44 (q, 4H), 2.61 (m, 4H), 2.55 (m, 4H), 1.85-1.82 (m, 4H), 1.60-1.58 (t, 6H). ^{13}C NMR (100 MHz, CD_3OD): δ 155.87, 154.00, 150.32, 143.97, 136.99, 133.11, 125.35, 124.75, 123.93, 119.83, 113.77, 112.64, 56.50, 30.74, 25.50, 21.81, 16.56. ESI-MS (m/z) 277.31 $[M/2]^{+}$. Solubility in water: ≈ 1.06 mM/L.

Synthesis of Compound 9 (DXBTZ). Compound 5 (86.5 mg, 0.25 mmol) and Compound 2 (182 mg, 0.625 mmol) were added into a tube. Then, anhydrous 1,2-dichlorobenzene (5 mL), acetic anhydride (3 mL) and piperidine (20 μL) were added into the tube. After sonication for 5 min, the tube was sealed and the mixture was stirred at 110 °C for 12 h. After cooling to room temperature, the solvent was evaporated in vacuum and the crude product was purified in a silica-gel column using dichloromethane/methanol (40:1 v/v) with 1% TFA as eluent. In order to be accurately weighed for the interaction with the host molecules, the purified product was further anion-exchanged by using tetrabutylammonium chloride to afford Compound 9. The product Compound 9 (DXBTZ) was finally obtained as a blue solid (48 mg, yield: 30%). ^{1}H NMR (400 MHz, DMSO-d_6): δ 8.25-8.23 (d, 2H), 8.08 (s, 2H), 8.05-8.04 (d, 2H), 7.71-7.67 (t, 2H), 7.65-7.61 (t, 2H), 7.33 (s, 2H), 7.19 (s, 2H), 7.01-6.98 (d, 2H), 4.18 (s, 6H), 2.74-2.71 (m, 4H), 2.63-2.60 (m, 4H), 1.86-1.80 (m, 4H). ^{13}C NMR (100 MHz, DMSO-d_6): δ 170.06, 155.23, 148.61, 141.95, 140.94, 131.73, 129.01, 127.69, 127.55, 126.83, 123.79, 123.73, 115.88, 113.50, 112.02, 108.08, 35.45, 28.93, 24.05, 19.78. ESI-MS (m/z) 319.34 $[M/2]^{+}$. Solubility in water: < 0.06 mM/L.

Changes in properties and ^{1}H NMR signals of guest molecules upon interaction with host molecule CB[8]. The absorbance, optoacoustic, fluorescence, photothermal heating trend and ^{1}H NMR signal changes of the guest molecule DXP (or DXBTZ) upon the addition of varied amounts of host molecule CB[8] in pure water (or water containing 10% DMSO) were recorded after 5 min of mixing at 25 °C. The absorption spectral variation was measured in a quartz cell (light path, 10 mm) on a UV-3600 Shimadzu UV-Vis-NIR Spectrophotometer. The optoacoustic

signal changes were measured by filling the test solutions in commercial Wilmad NMR tubes and then acquiring the data on a multispectral optoacoustic tomographic imaging system (inVision 128, iThera Medical GmbH). The alteration of fluorescence intensity was measured by adding the test solutions into six-well plates and then recording the data by using Living Image 4.3 software of an IVIS Spectrum imaging system. The fluorescence intensity of DXP at 760 nm upon the addition of varied amounts of CB[8] was recorded with an excitation wavelength of 640 nm, while that of DXBTZ at 820 nm after adding different amounts of CB[8] was measured with an excitation wavelength of 675 nm. The photothermal heating trend was attained by monitoring the changes in temperature of the solutions of CB[8] alone, DXP (or DXBTZ) alone or DXP (or DXBTZ) complexed with CB[8] upon the irradiation of 730 nm laser (0.5 W cm^{-2}). The temperature of the samples was recorded by an electronic thermometer. For the changes in ^{1}H NMR signals, data were collected on a Bruker BBFO 400 Spectrometer.

Quantum chemical calculations. All the simulations were performed by using the Gaussian 16_A01 program package.[1] The geometry optimizations of DXP and DXBTZ were conducted using density functional theory (DFT) in B3LYP-(D3BJ)/TZVP level.[2] The optimized structures were confirmed without observing imaginary frequency. Excited state energies were calculated in M06-2x/6-31G* level in DMSO solution employing polarized continuum model (PCM). HOMO and LUMO plots were visualized using VMD 1.9.3 software.[3]

Cell culture. Human embryonic kidney normal cells (HEK 293), human breast adenocarcinoma epithelial cells (MDA-MB-231), mouse fibroblast cell line (L929) and mouse mammary adenocarcinoma cell line (4T1) were obtained from American Type Culture Collection (ATCC). Human fetal hepatocyte line (L-02) and human bladder cancer cell line (T24) were purchased from Ubigene Biosciences Co., Ltd. T24 cells was incubated in RPMI-1640 medium supplemented with 10% CS (calf serum, GIBCO) and 1% penicillin and streptomycin at 37 °C under 5% of CO2, while the other five cell lines were cultured in Dulbecco's modified eagle medium (DMEM) supplemented with 10% FBS and 1% penicillin and streptomycin at 37 °C under 5% of CO_2. When the cell density reached 70-80% of confluence, subculturing was considered complete. The medium was changed approximately

every 1-2 days.

Cytotoxicity studies. The relative viabilities of HEK 293, L929, L-02, 4T1, T24 and MDA-MB-231 cells which were exposed to DXBTZ-CB[8]/CSA were assessed by MTT assay. These cell lines were seeded in 96-well plates with an initial seeding density of 5×10^4 per milliliter and cultured in the medium. After a 24-h period of incubation at 37 °C under 5% of CO_2, the cells were washed with pre-warmed PBS solution, and then PBS was substituted with fresh culture medium containing the nanoagent DXBTZ-CB[8]/CSA (concentration: 0, 10, 20, 30, 40, or 50 μg mL^{-1}), after that the cells were subject to incubation for 24 h. Afterwards, the wells were washed with PBS and incubated for another 4 h with medium containing 0.5 mg mL^{-1} MTT. Upon discarding the culture medium, DMSO (150 μL) was added to dissolve the precipitates and the absorbance was later measured with a Tecan's Infinite M200 microplate reader at 570 nm. In the control experiments, the cells were treated with the same concentrations of DXBTZ-CB[8]/CSA and incubated at the same condition, but no MTT was added. After adding the same volume of DMSO, the absorbance of control groups was also measured at 570 nm. The absorbance subtraction at 570 nm between the groups with or without addition of MTT was recorded to eliminate the intrinsic absorption of DXBTZ-CB[8]/CSA. Finally, the statistical mean and standard deviation of absorbance subtraction at 570 nm were employed to estimate the relative cell viability. In the assays, for each concentration, three independent experiments were performed.

Cellular uptake of the nanoagent DXBTZ-CB[8]/CSA. The cellular uptake of the nanoagent DXBTZ-CB[8]/CSA was evaluated by flow cytometry and changes in optical density at 692 nm (OD_{692}). For flow cytometry, the CD44-overexpressed MDA-MB-231 cancer cells were seeded onto six-well plates at 2×10^5 cells per milliliter and allowed to culture for 24 h before treatments. Except for cells from the control well (0 min), the cells in the other five wells were incubated with DXBTZ-CB[8]/CSA nanoparticles (final concentration: 50 μg mL^{-1}) for different time (15, 30, 45, 60 and 90 min). Afterwards, the cells from different wells were washed, trypsinized, centrifuged and then resuspended in PBS for flow cytometry. On a BD FortessaX20 5-laser cytometer, the cells from different wells were first identified through FSC-

A vs. SSC-A to remove debris and free nanoparticles, and then single cells were further identified through FSC-A vs. FSC-H. Finally, the fluorescence intensity of 10,000 cells from each well were recorded and analyzed.

As for the measurement of cell optical density at 692 nm (OD_{692}) following uptake of DXBTZ-CB[8]/CSA nanoparticles, MDA-MB-231 cells were first seeded onto 96-well plates at 5×10^4 cells per milliliter, and allowed to adhere after 24 hours of culture. Then, DXBTZ-CB[8]/CSA nanoparticles (final concentration: 50 μg mL^{-1}) were added in each well except the control wells (0 min). After incubated for different time (15, 30, 45, 60 and 90 min), the cells were washed three times with PBS to remove free DXBTZ-CB[8]/CSA nanoparticles in the medium. Finally, OD_{692} of cells in each well was measured with a microplate reader. For each incubation time, optical density measurement was performed on five independent cell wells.

Cell imaging. HEK293, L929 and L-02 normal cells and CD44-overexpressed 4T1, T24 and MDA-MB-231 cancer cells were seeded with a density of 5×10^4 per milliliter in a confocal dish and allowed to adhere. After 1 day, the cells were washed with PBS, and then incubated in PBS containing the nanoagent DXBTZ-CB[8]/CSA (50 μg mL^{-1}) at 37 °C under 5% CO_2. After 60 min, the cells were washed with PBS and then incubated with Hoechst 33342 (10 μg mL^{-1}) in PBS for 10 min. To verify the CD44 targeting ability of DXBTZ-CB[8]/CSA nanoparticles, some MDA-MB-231 cells were pre-treated with CD44 inhibitor hyaluronic acid (5 mg mL^{-1}) or anti-CD44 antibody ([catalog No.:1M7.8.1] Abcam, 10 μg mL^{-1}) for 30 min. Before subjected to fluorescence imaging, the culture dishes were washed with PBS for three times. As for multispectral optoacoustic imaging of cellular uptake, the cells in culture dishes were washed, trypsinized, centrifuged, suspended in PBS, and fully filled into commercial Wilmad NMR tubes, and then fixed on the holder of the imaging instrument for data collection.

Animal experiments. The male BALB/c mice and female BALB/c nude mice (5-6 weeks old) were purchased from Guangdong Medical Laboratory Animal Center (GDMLAC, China) or from InVivos Pte. Ltd. (Singapore). The animals were housed in sterile cages within laminar airflow hoods at 24 °C, 45-65% humidity in a specific pathogen-free room with a 12 h light/12 h dark schedule and fed autoclaved chow and water ad libitum. Mice were randomly allocated

to different groups to carry out subsequent experimental investigations. In the case of lethal experimental procedures, mice would be euthanized by exposure to carbon dioxide gas in a rising concentration.

Mouse model of subcutaneous tumor. For the mouse model of subcutaneous tumor, 2×10^6 MDA-MB-231 cancer cells suspended in a Matrigel/PBS (3:2) mixture (60 μL) were subcutaneously injected onto the back of female BALB/c nude mice and incubated for 2 weeks before imaging.

Mouse model of orthotopic bladder tumor. Chemical lesions to the bladder urothelium of female nude mice were performed by injecting silver nitrate (10 μL, 0.5 M) into the bladder of each animal through a 24-gauge catheter for 10 s. Bladders were washed with PBS and subsequently instilled with T24 cells (5×10^5) suspended in PBS (50 μL) via the catheter. Cells were retained in the bladder for 2 h by tying off the orifice of the urethra. Experiments with tumor-bearing mice were performed 20 days post inoculation of T24 cells.

Mouse model of lymphatic metastasis of tumor. For development of a lymph node metastasis mouse model, 2.5×10^5 4T1 cells suspended in PBS (pH 7.4, 20 μL) were injected into the right hind footpads of female nude mice and incubated for 21 days before experiments.

Mouse model of renal ischemia/reperfusion injury. For the renal ischemia/reperfusion injury mouse model, male mice were anesthetized and placed on a thermostatically controlled heating pad to maintain body temperature (Step 1). An abdominal midline incision was made to expose the kidneys (Step 2) and the bilateral renal pedicles were clamped with the nontraumatic microvascular clamps (Step 3). The occlusion was confirmed visually by change in the kidneys' color from bright red to dark purple. After 60 min of ischemia, the clamps were released and the kidneys' color returned to red (Step 4). Finally, the incisions were closed using a sterile 6-0 biodegradable surgical silk suture (Step 5). After reperfusion for 24 h, the mice were subjected to experiments (Step 6). As for the sham-operated control group, the mice underwent the surgical procedures without clamping of renal pedicles and acted as controls.

Biosafety evaluation. The biosafety of DXBTZ-CB[8]/CSA nanoagents was evaluated by histological examination, body weight measurement, blood routine tests and serum biochemical assays. The mice administrated with PBS buffer (control) or the nanoagent DXBTZ-CB[8]/CSA dispersion (62.2 mg kg^{-1}) were weighed every day and sacrificed 7 days post injection. Then, the blood samples and major organs of these mice were collected and analyzed. For blood routine tests, parameters such as hemoglobin (HGB), white blood cells (WBC), red blood cells (RBC), hematocrit (HCT), corpuscular hemoglobin (MCH), mean corpuscular volume (MCV), mean corpuscular hemoglobin concentration (MCHC), red cell distribution width (RDW), and platelets (PLT) were measured. For serum biochemical assays, alanine aspartate aminotransferase (AST), transaminase (ALT), blood urea nitrogen (BUN), and creatinine (CREA) indicating hepatic and renal function were measured. As for histological examination, the collected organs were embedded in paraffin, sectioned to 4 μm for hematoxylin and eosin (H&E) staining and then observed under a microscope.

Immunofluorescence staining. The excised tissues were embedded in Tissue-Tek OCT compound and snap frozen in liquid nitrogen. Then, the frozen tissues were cut into 15 μm sections. Afterwards, the frozen sections were incubated with primary antibody (anti-CD44 [catalog NO.: A16807] ABclonal) at a 1:100 dilution, followed by an Alexa Fluor 488-labeled secondary antibody (Goat Anti-Rabbit IgG H&L [catalog NO.: ab150077] Abcam) at a 1:100 dilution and then visualized under a confocal microscope. Final images were pseudo-color-coded blue for DAPI (nuclei) and red for target protein (CD44 receptors).

Immunohistochemistry staining. In brief, the slides of excised tissues were deparaffinized and rehydrated in PBS. Then, endogenous peroxidase activity was quenched by immersion in 3% H_2O_2 in methanol for 10 min. A nonspecific blocking procedure making use of 5% bovine serum albumin was performed before application of primary antibodies. The antibody (anti-CD206 [catalog NO.: ab300621] abcam or anti-IL-6 [catalog NO.: EM1701-45] HuaBio) was used at a 1:50 dilution. The incubation was performed at 4 °C overnight. Then, an Elivision™ plus Polyer HRP (Mouse) IHC Kit was used to amplify the signal of antigen-antibody binding

according to the manufacturer's instructions. The slides were colorized with fresh DAB solution and washed in water to end the reaction. After dried overnight, the slides were counterstained with hematoxylin, cover-slipped and observed under an optical microscope.

Western blot analysis. Kidney tissues were harvested and rinsed in cold sterile PBS and then homogenized in ice-cold RIPA Lysis Buffer (1/10, w/v, Keygen Biotech) with protease inhibitor cocktails (0.1 %) and PMSF (1 mM, Keygen Biotech). Homogenates were centrifuged at 12000 g for 5 min at 4 °C, and supernatants were assayed for protein content using a BCA Protein Assay Kit (Keygen Biotech) according to instructions provided. SDS-PAGE loading buffer was added into the protein supernatants (1/4, v/v, Keygen Biotech) and then the mixture was boiled for 10 min (100 °C). After brief centrifugation, the protein samples were stored at -20 °C until further use. The protein samples were separated by electrophoresis on SDS-PAGE gels and transferred to PVDF membranes. The PVDF membranes were blocked with the Western Blocking Buffer (Keygen Biotech) at room temperature for 1 h. After washed by PBST three times, the membranes were incubated with primary antibodies (anti-MMP-2 [catalog No.: ET1606-4] HuaBio, anti-MMP-9 [catalog No.: A11147] ABclonal, or β-actin [catalog No.: BS6007M], Bioworld) at 1:1000 dilution under 4 °C overnight. After washed by TBST 5 times, the membranes were incubated with HRP-conjugated secondary antibodies ([catalog No.: BS13278, BS12478], Bioworld) at 1:5000 dilution for 1 h. After washed by TBST 5 times, the membranes were subject to chemiluminescence detection (ECL Kit, Keygen Biotech) by Tanon 5200 Imaging System.

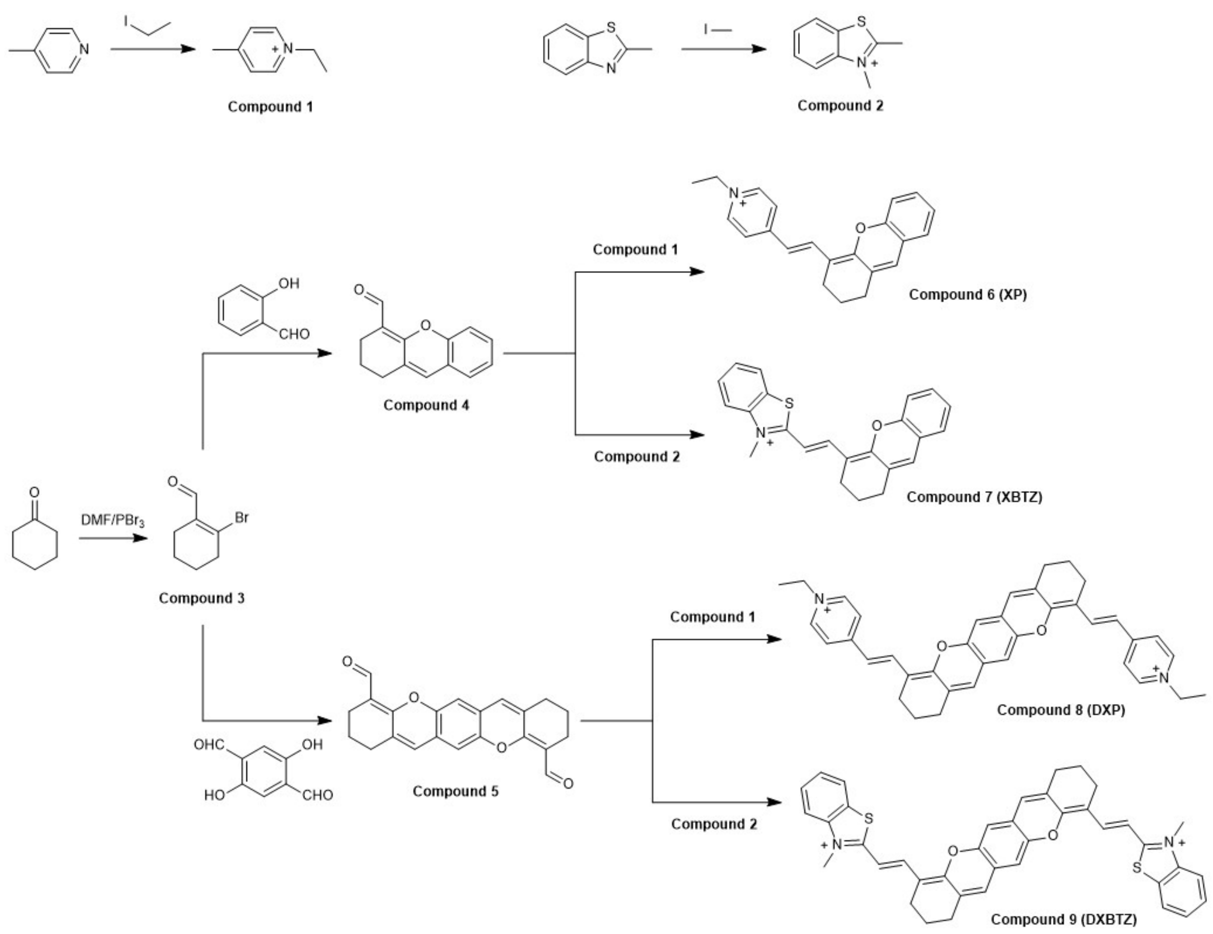

Supplementary Figure 1. Synthesis route of the model organic chromophores DXP and DXBTZ, and their corresponding control compounds XP and XBTZ.

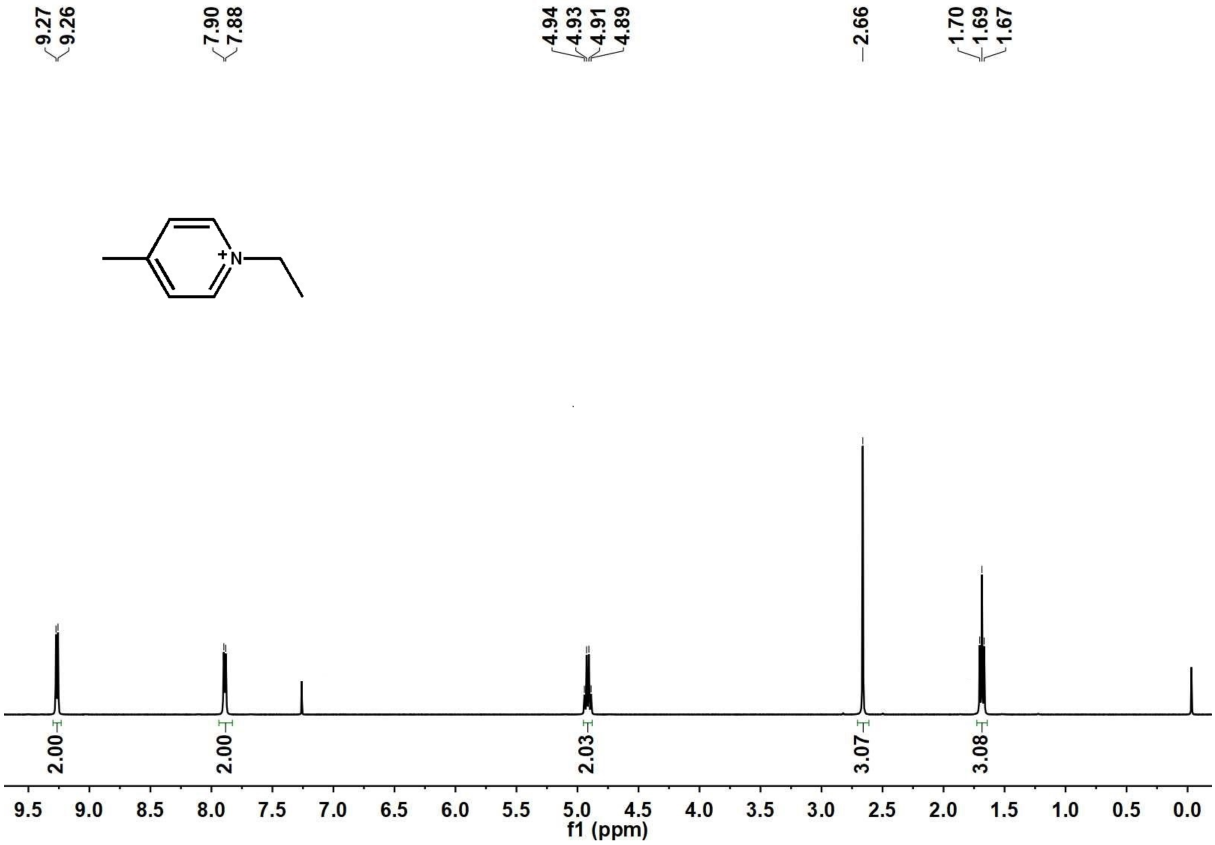

Supplementary Figure 2. ^{1}H NMR spectrum of Compound 1 in $CDCl_3$.

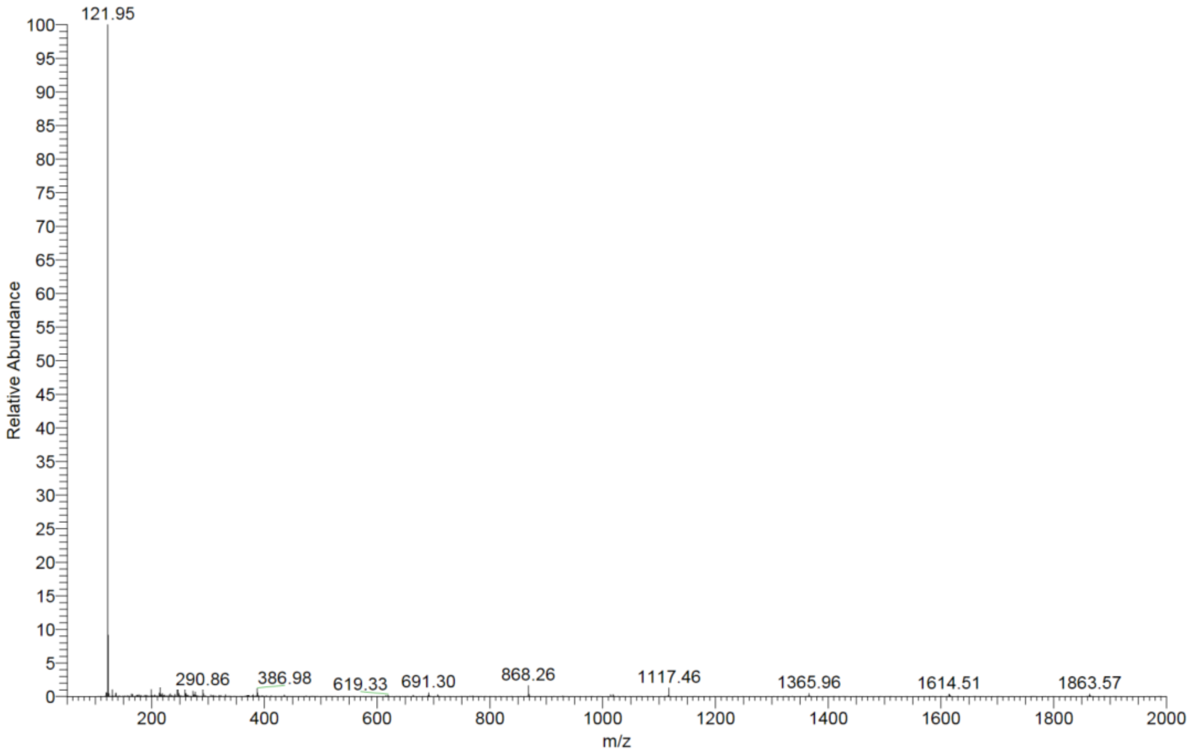

Supplementary Figure 3. Mass spectrum of Compound 1. MS (ESI): *m/z* 121.95 $[M]^{+}$.

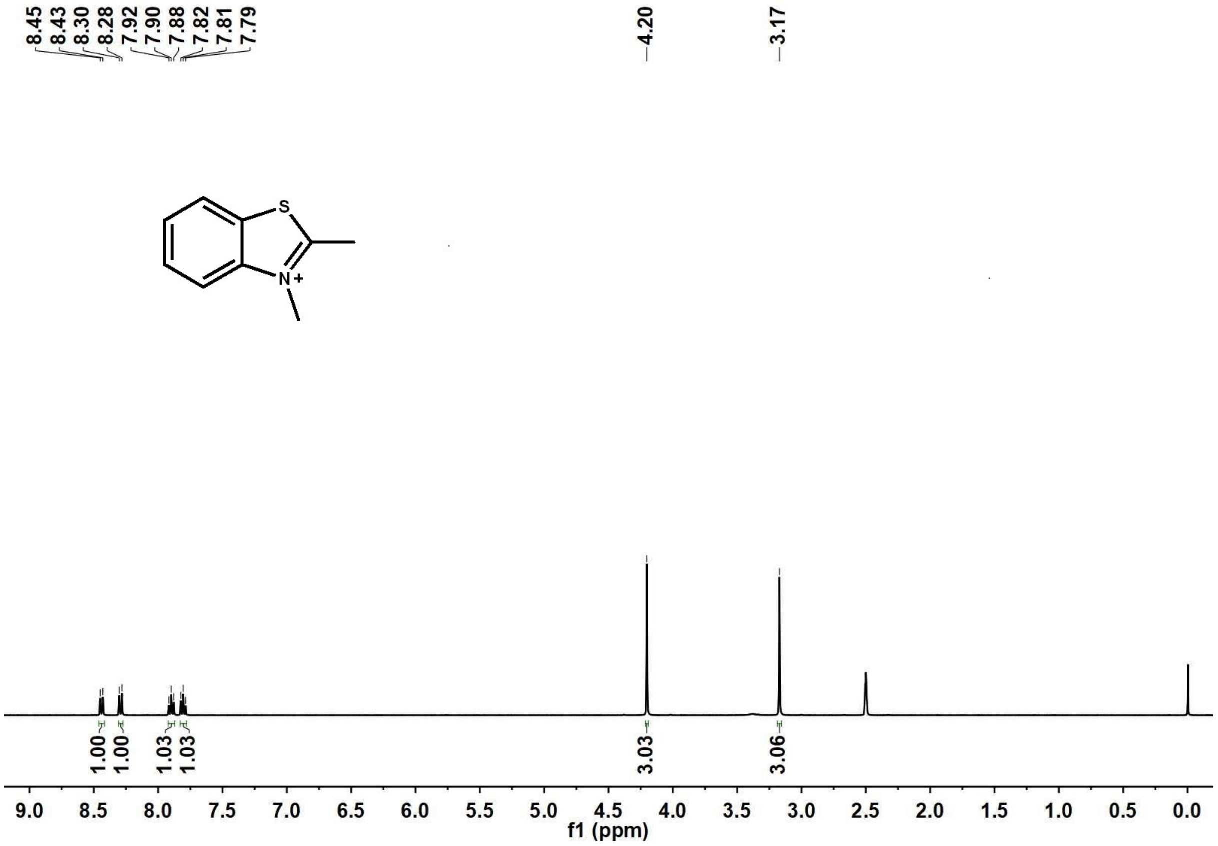

Supplementary Figure 4. ^{1}H NMR spectrum of Compound 2 in DMSO-d_6.

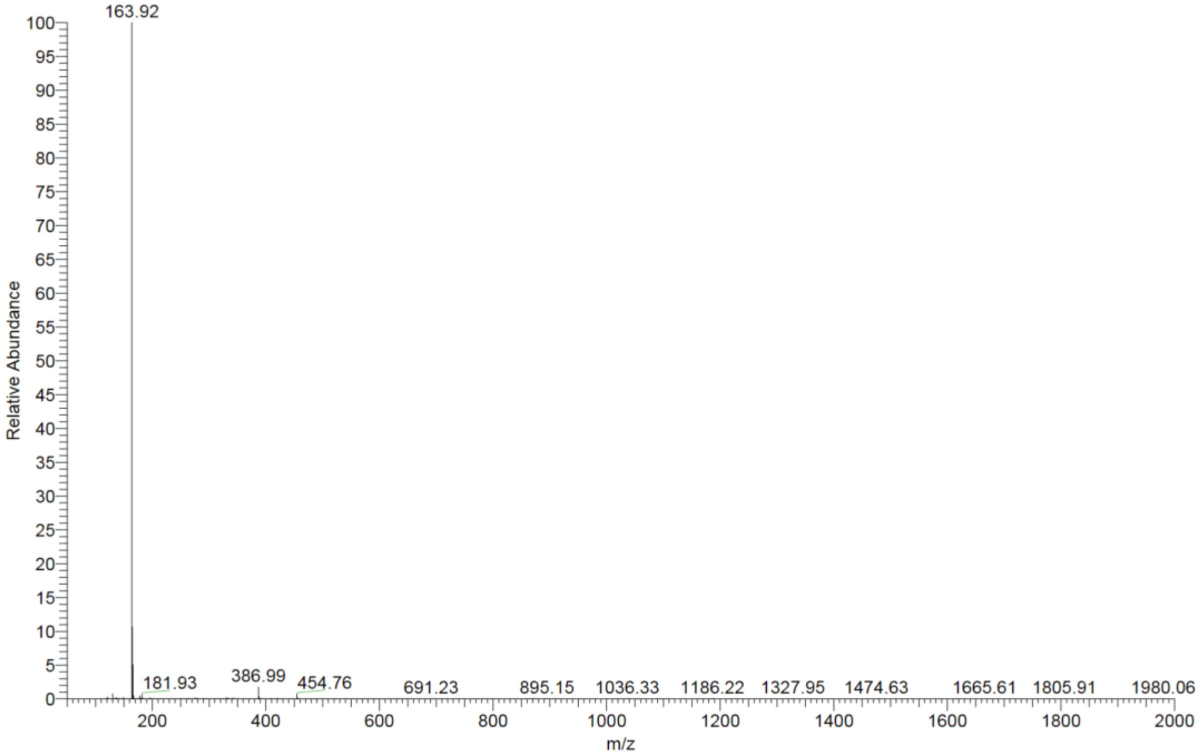

Supplementary Figure 5. **Mass spectrum of Compound 2.** MS (ESI): *m/z* 163.92 $[M]^{+}$.

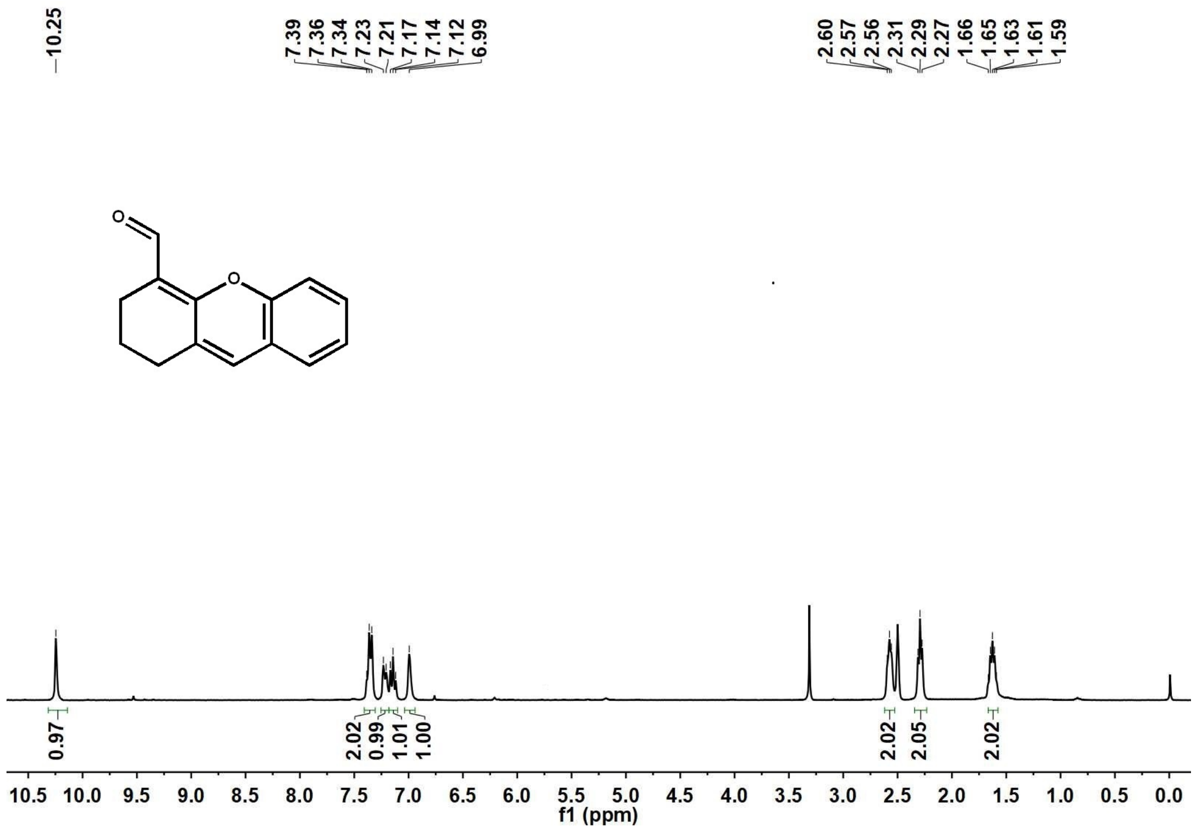

Supplementary Figure 6. ^{1}H NMR spectrum of Compound 4 in DMSO-d_6.

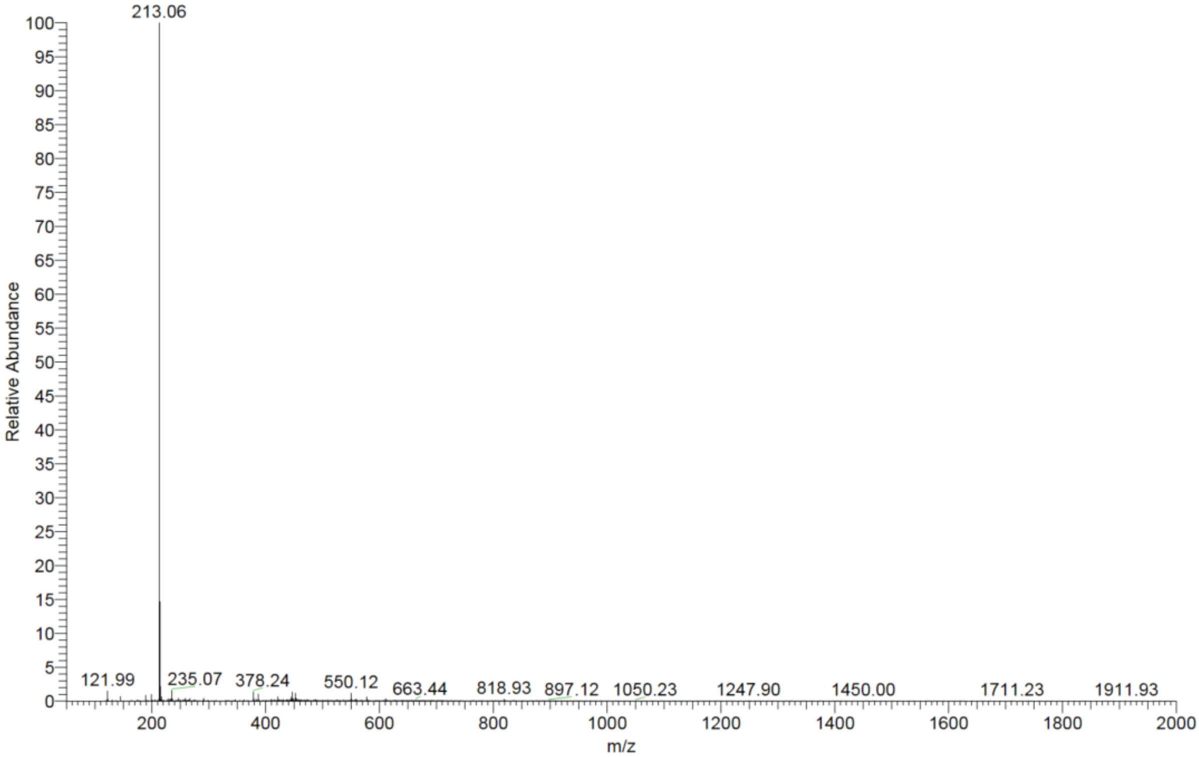

Supplementary Figure 7. Mass spectrum of Compound 4. MS (ESI): *m/z* 213.06 $[M+H]^+$.

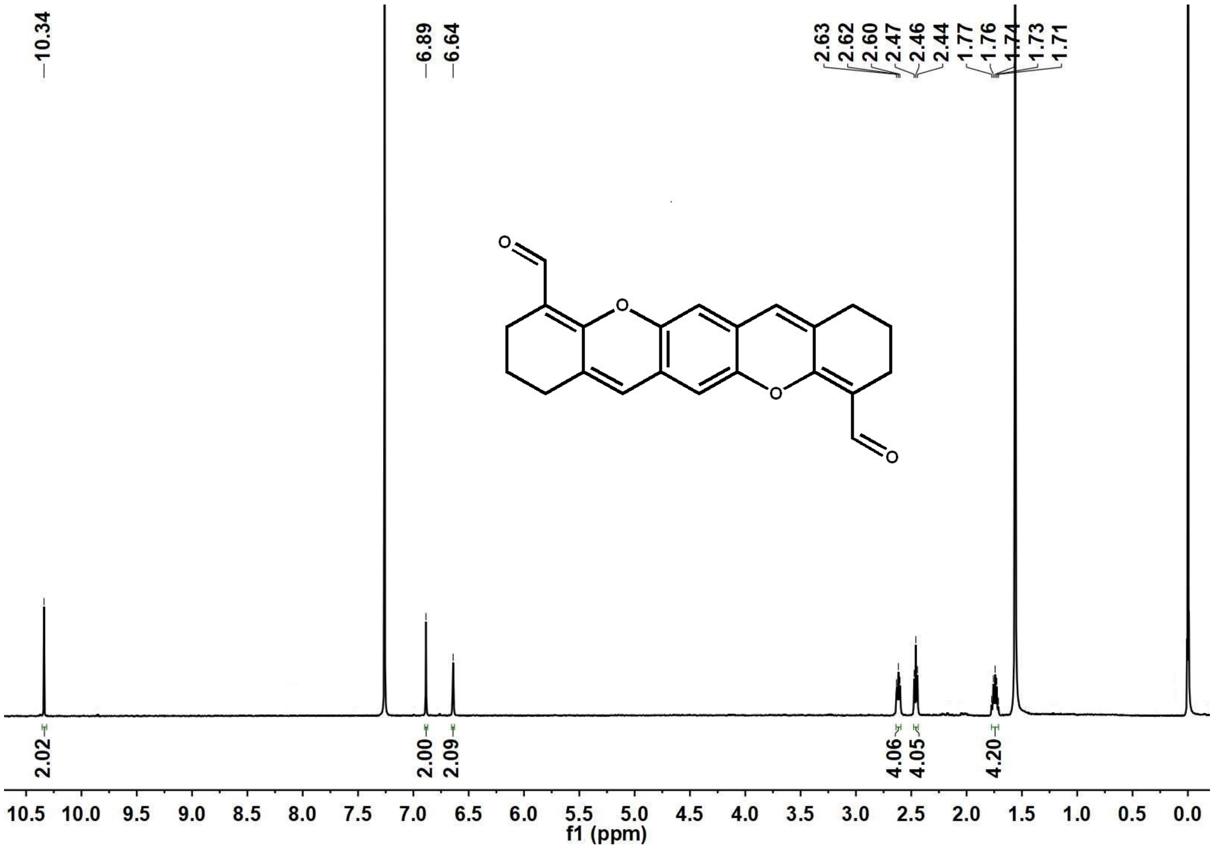

Supplementary Figure 8. ^{1}H NMR spectrum of Compound 5 in $CDCl_3$.

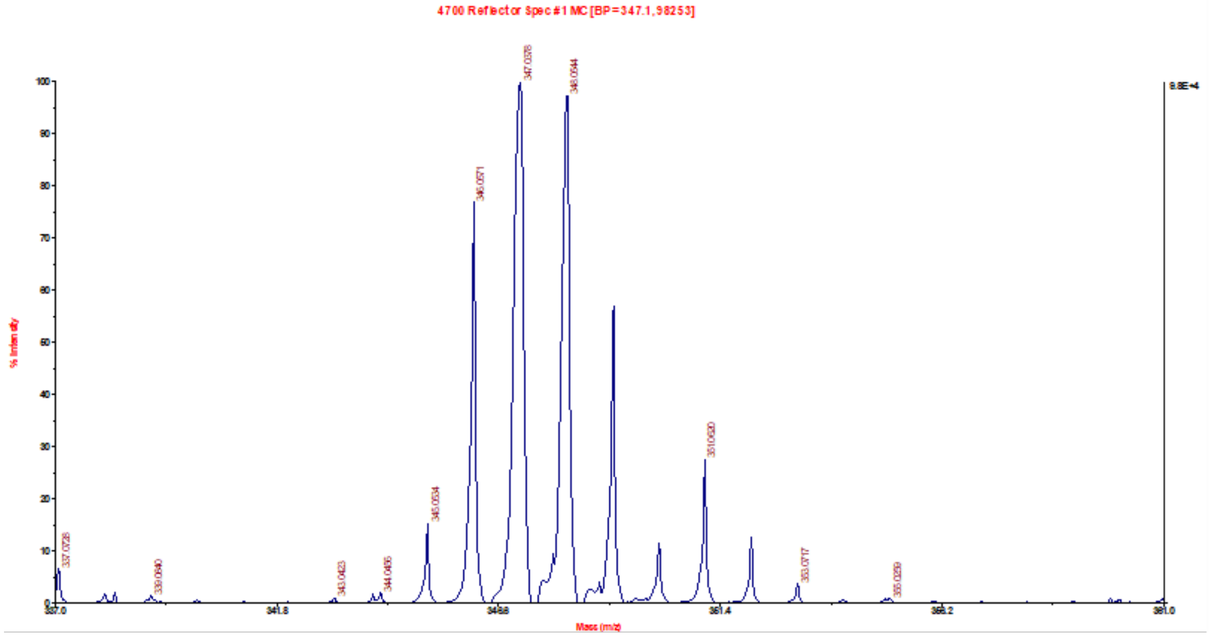

Supplementary Figure 9. MALDI-TOF mass spectrum of Compound 5.

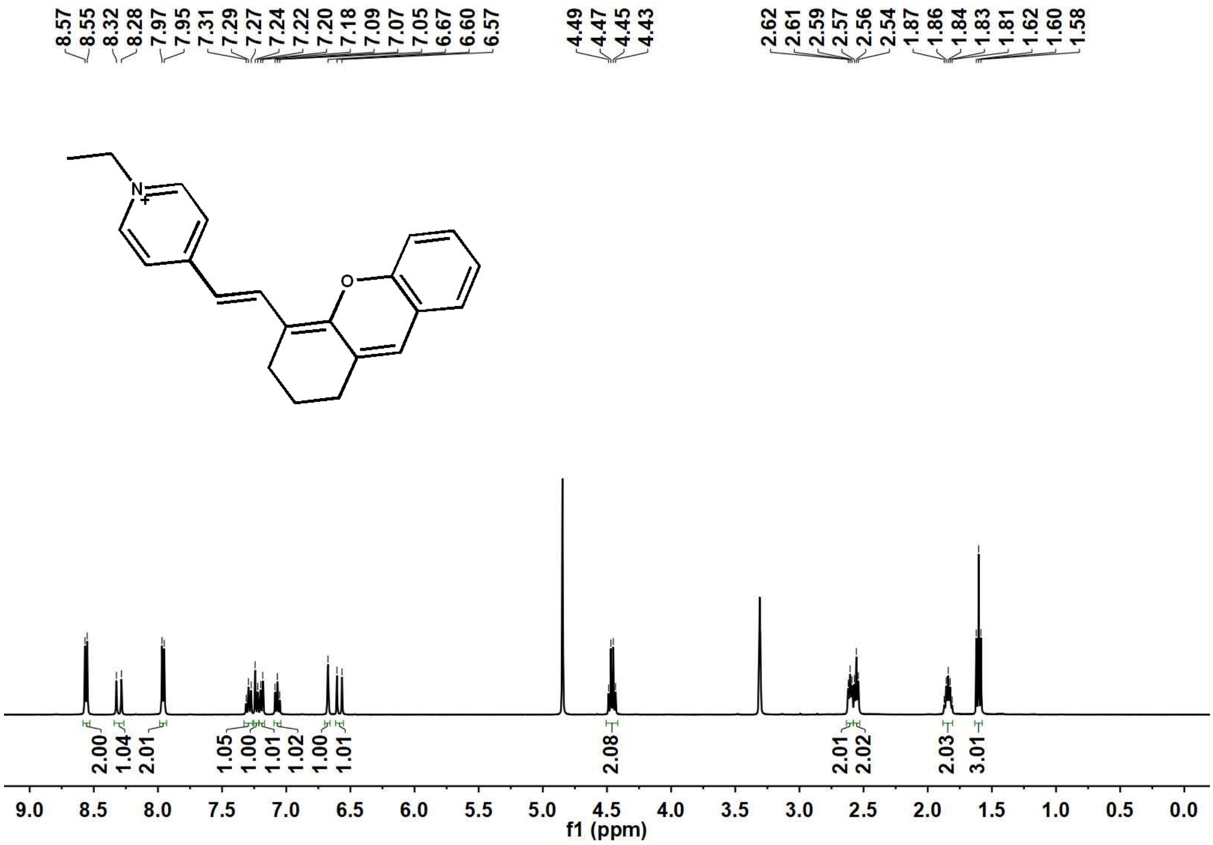

Supplementary Figure 10. ^{1}H NMR spectrum of Compound 6 (XP) in MeOD-d_4.

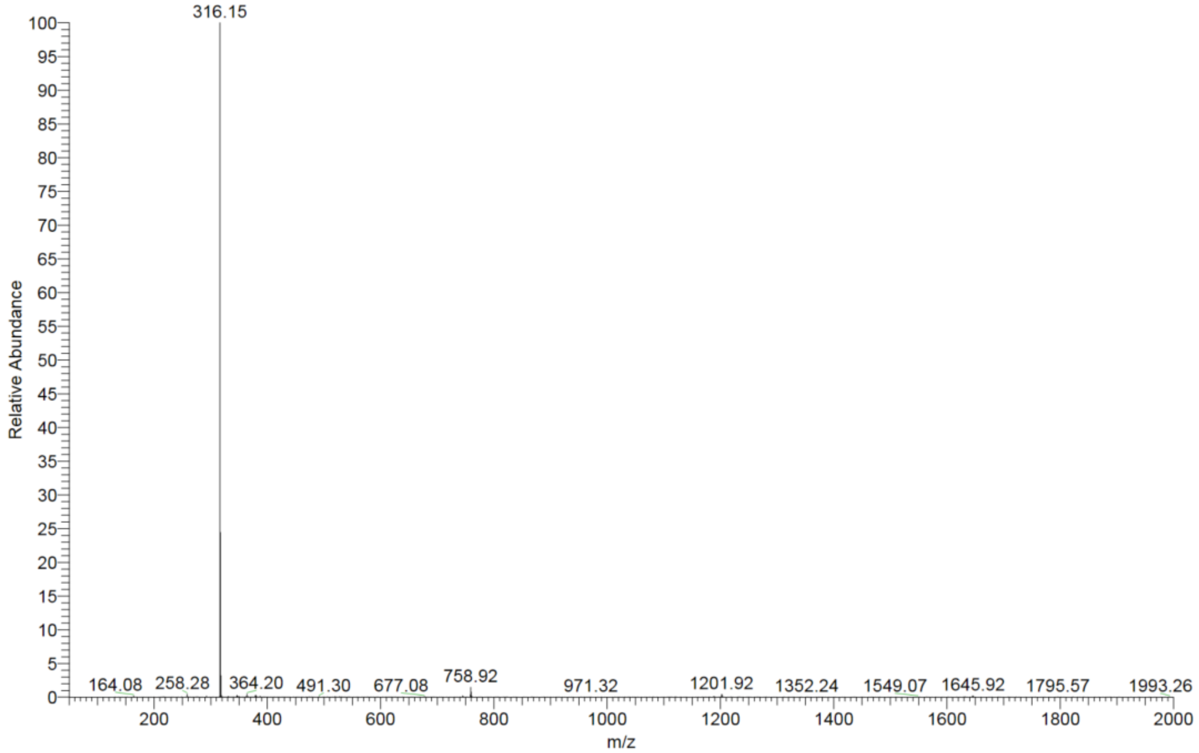

Supplementary Figure 11. Mass spectrum of compound 6 (XP). MS (ESI): *m/z* 316.15 $[M]^+$.

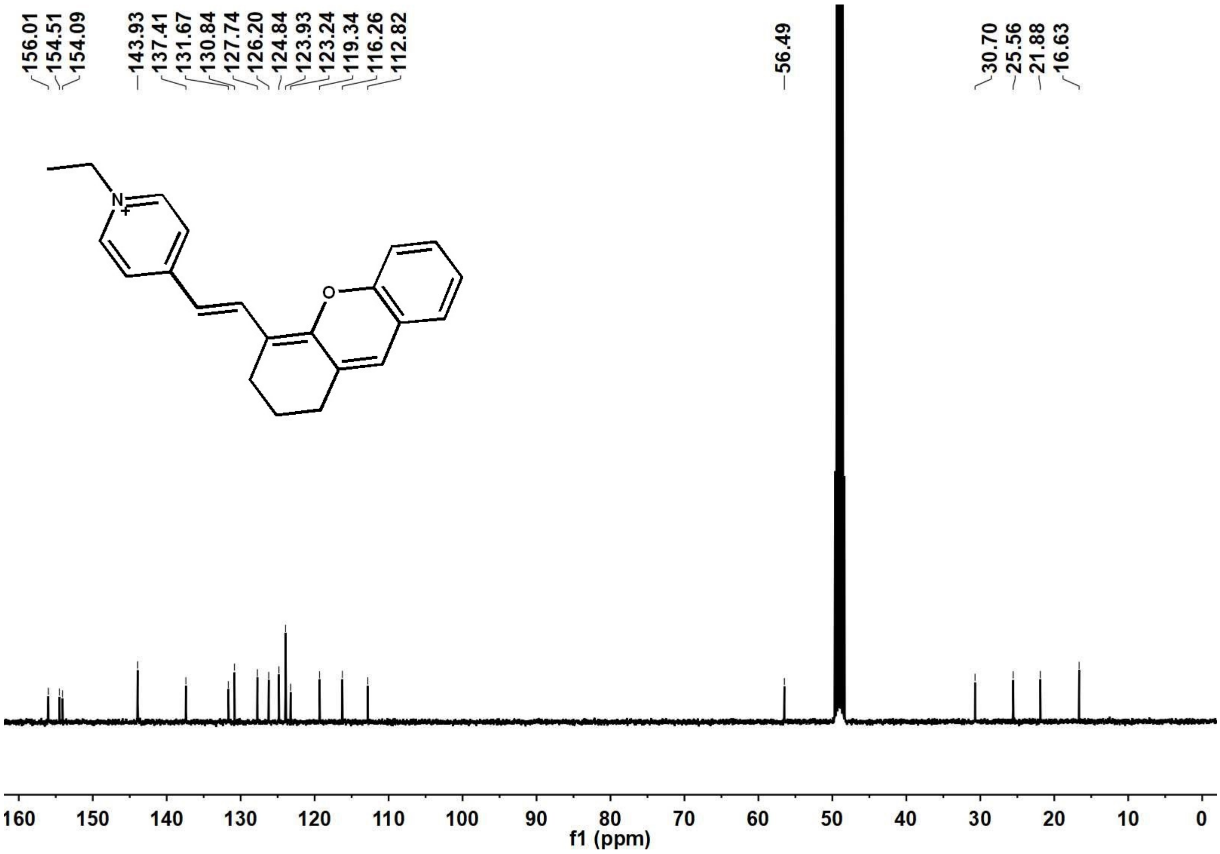

Supplementary Figure 12. ^{13}C NMR spectrum of Compound 6 (XP) in MeOD-d_4.

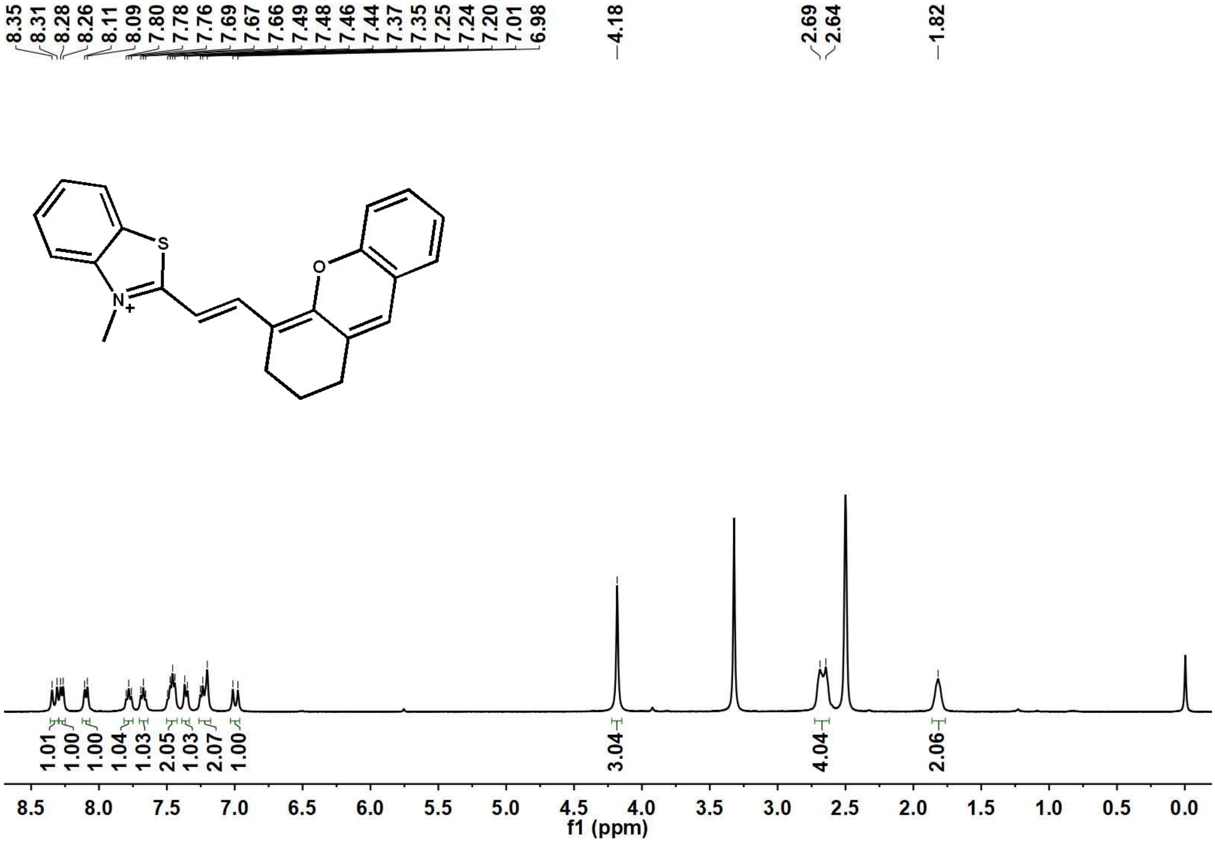

Supplementary Figure 13. ^{1}H NMR spectrum of Compound 7 (XBTZ) in DMSO-d_6.

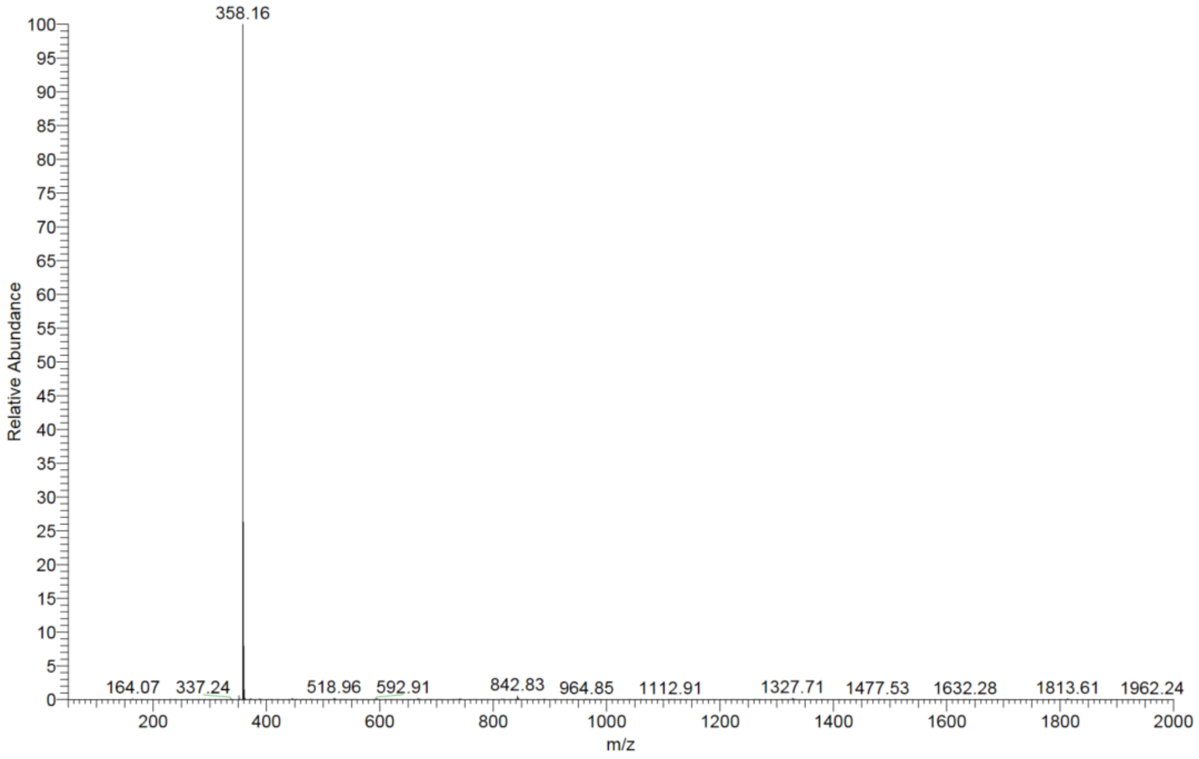

Supplementary Figure 14. Mass spectrum of Compound 7 (XBTZ). MS (ESI): *m/z* 358.16 $[M]^+$.

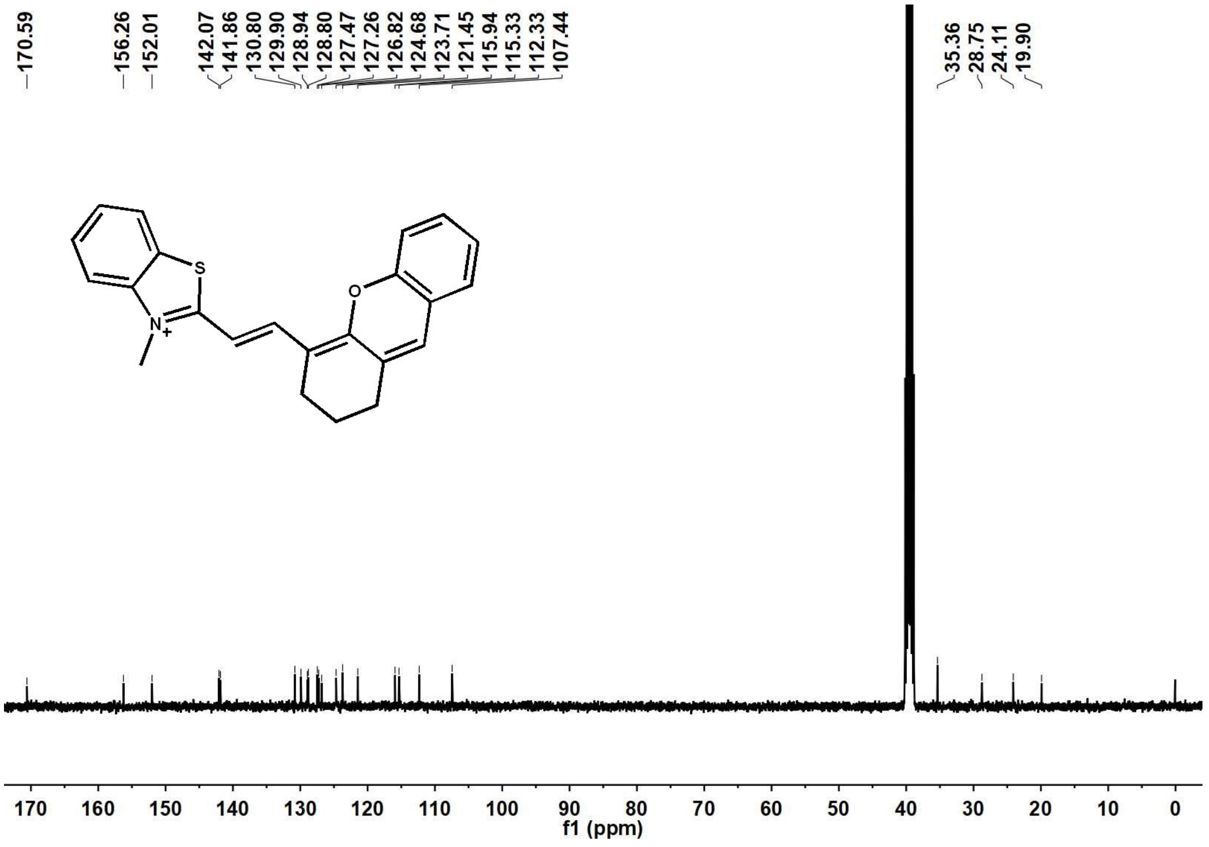

Supplementary Figure 15. ^{13}C NMR spectrum of Compound 7 (XBTZ) in DMSO-*d*$_6$.

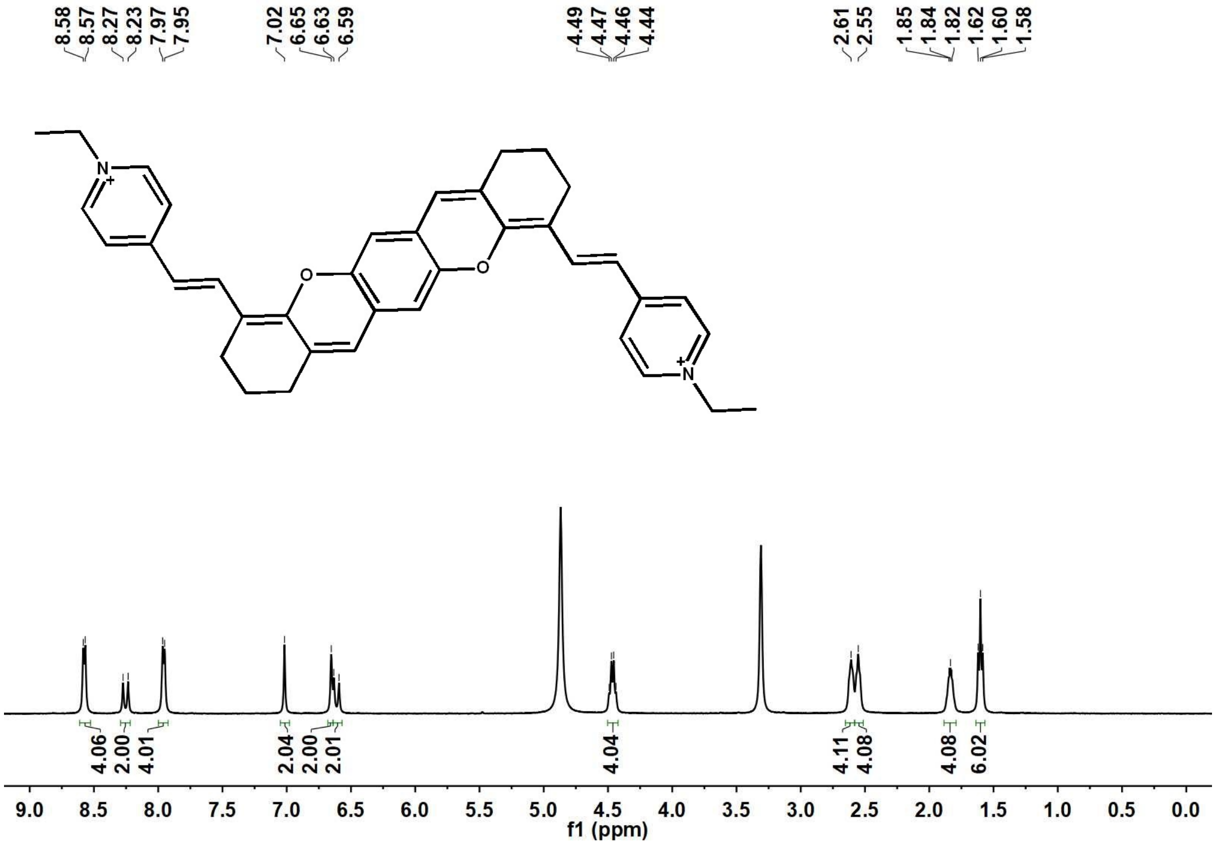

Supplementary Figure 16. ^{1}H NMR spectrum of Compound 8 (DXP) in MeOD-d_4.

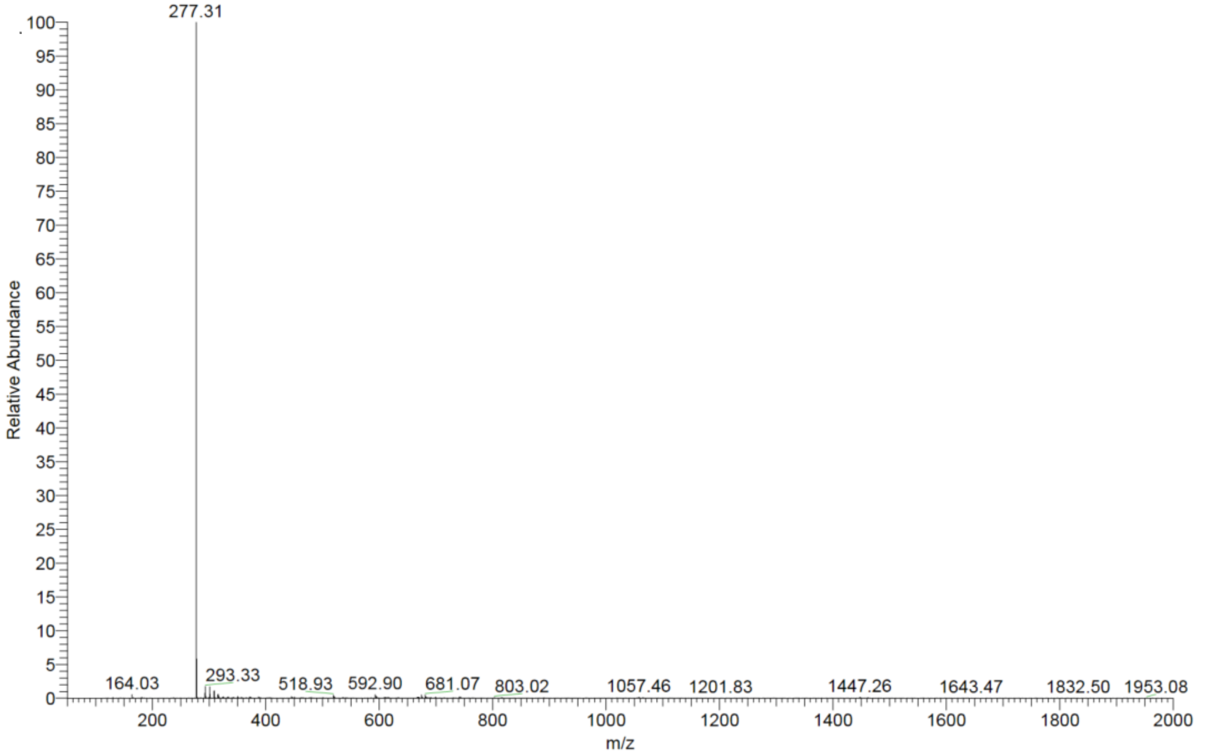

Supplementary Figure 17. Mass spectrum of Compound 8 (DXP). MS (ESI): *m/z* 277.31 $[M/2]^{+}$.

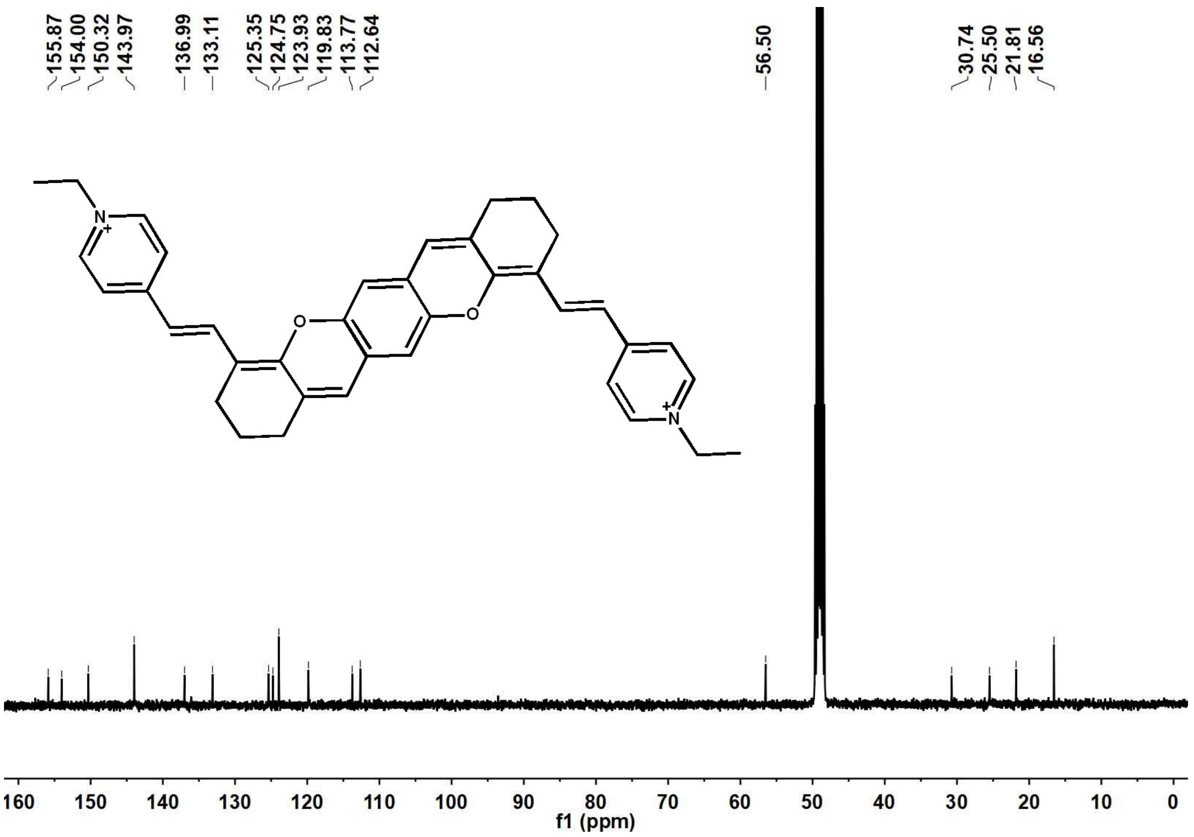

Supplementary Figure 18. ^{13}C NMR spectrum of Compound 8 (DXP) in MeOD-d_4.

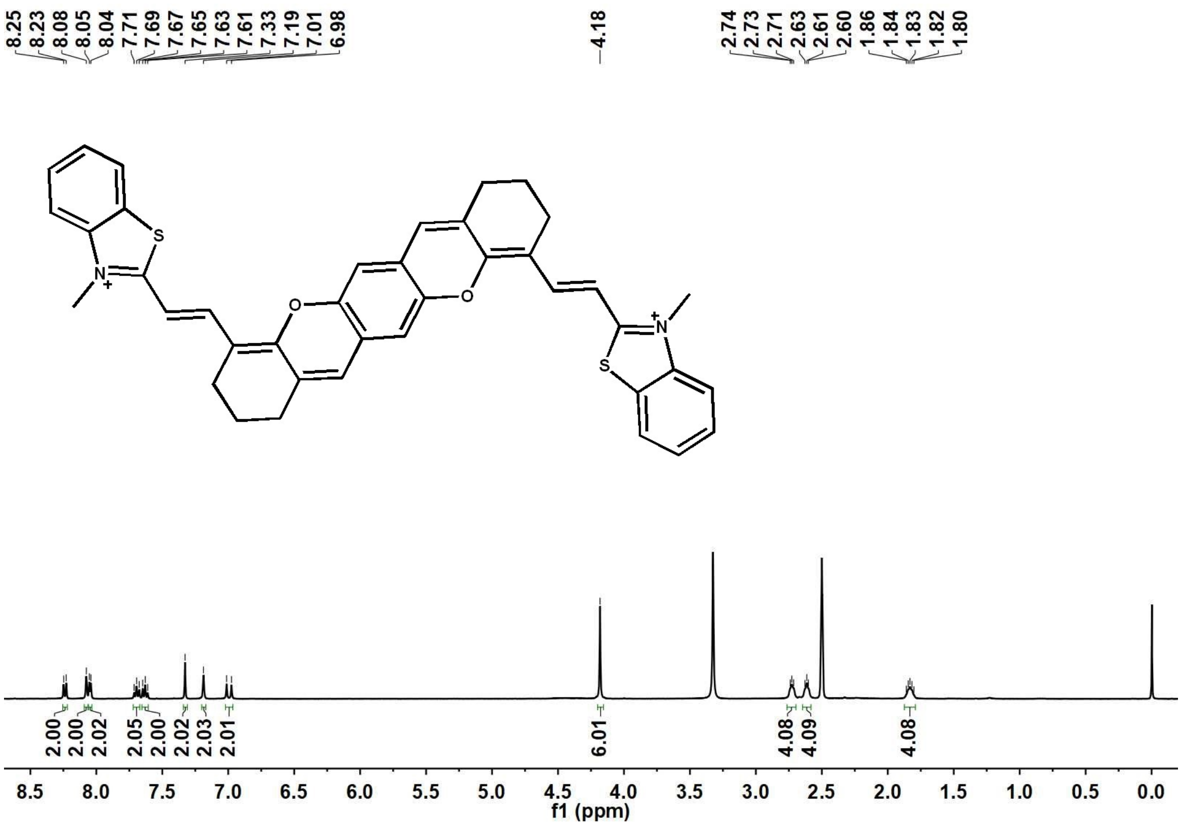

Supplementary Figure 19. ^{1}H NMR spectrum of Compound 9 (DXBTZ) in DMSO-d_6.

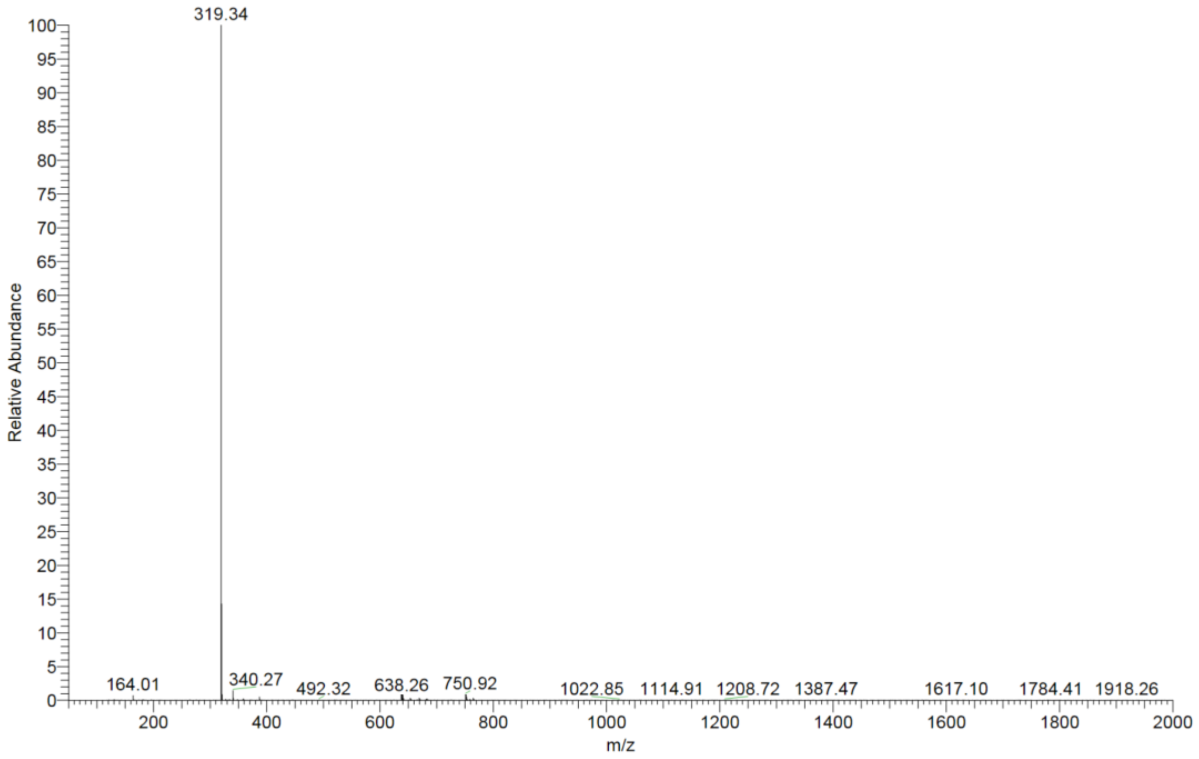

Supplementary Figure 20. Mass spectrum of Compound 9 (DXBTZ). MS (ESI): *m/z* 319.34 $[M/2]^{+}$.

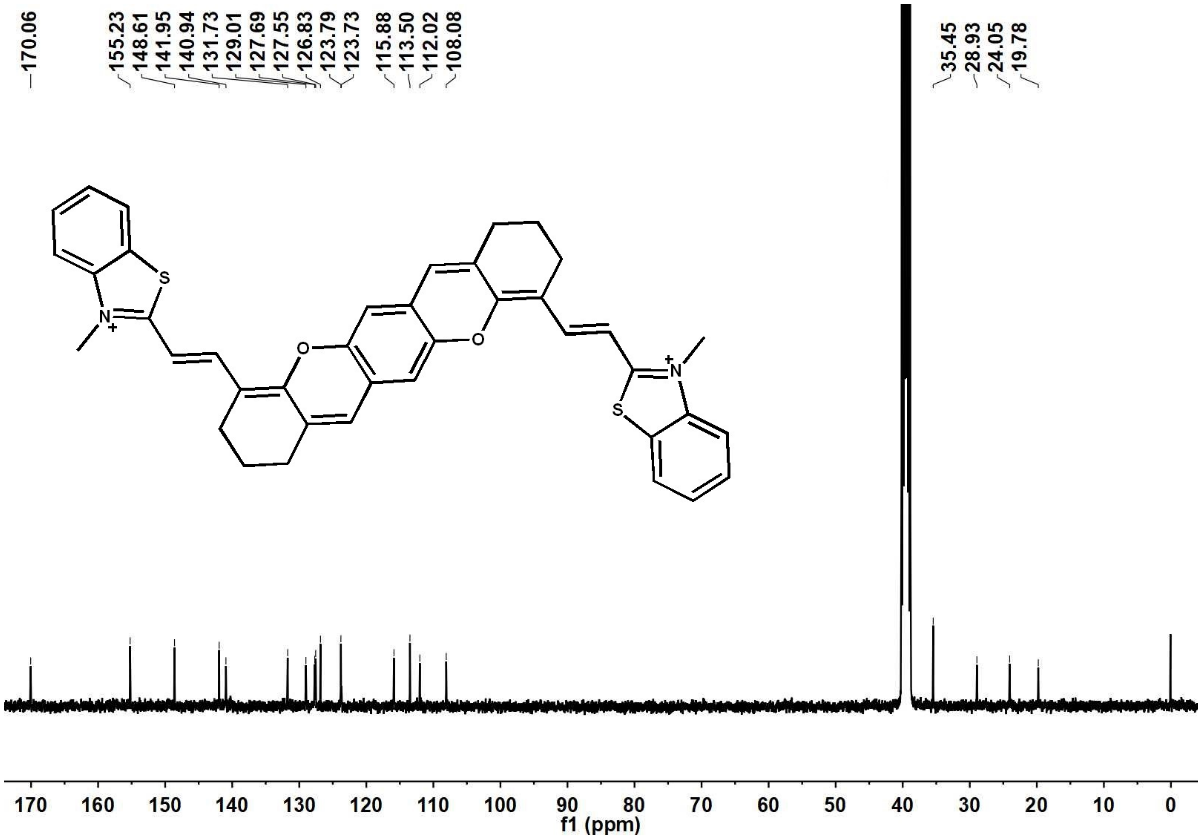

Supplementary Figure 21. ^{13}C NMR spectrum of Compound 9 (DXBTZ) in DMSO-*d*$_6$.

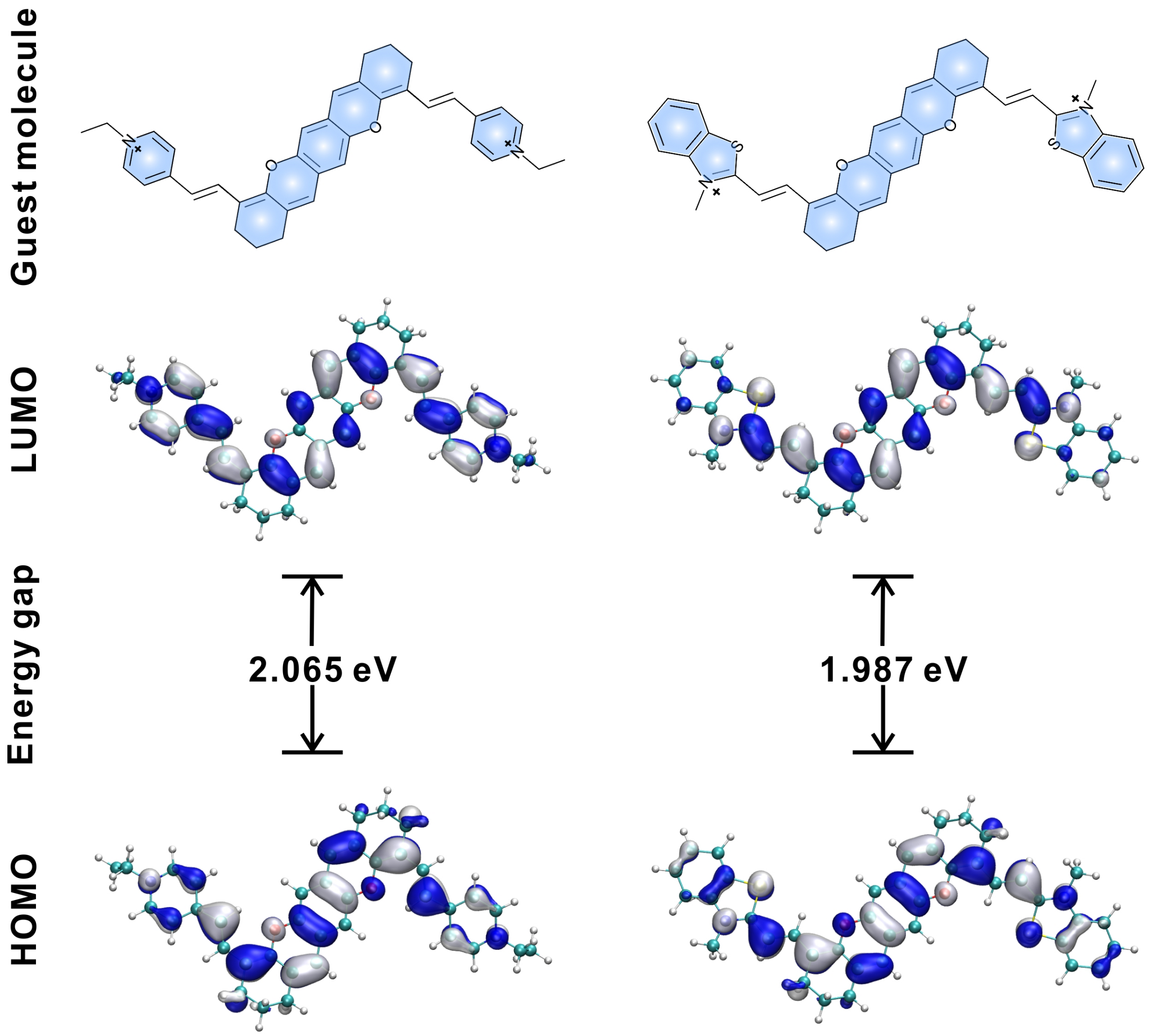

Supplementary Figure 22. Theoretical calculations. HOMO and LUMO orbital surfaces of DXP and DXBTZ in the geometrically optimized structures (Fig. 3b and 3c). LUMO: lowest unoccupied molecular orbital; HOMO: highest occupied molecular orbital.

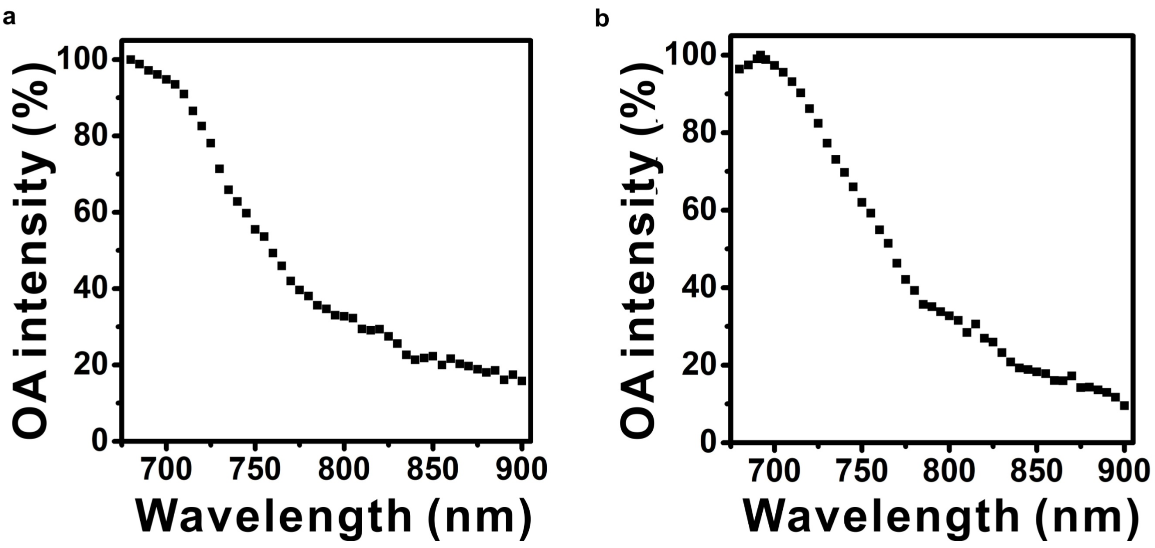

Supplementary Figure 23. Optoacoustic spectrum. Wavelength-dependence of relative OA intensity for (a) DXP and (b) DXBTZ after mixing with equimolar amount of CB[8].

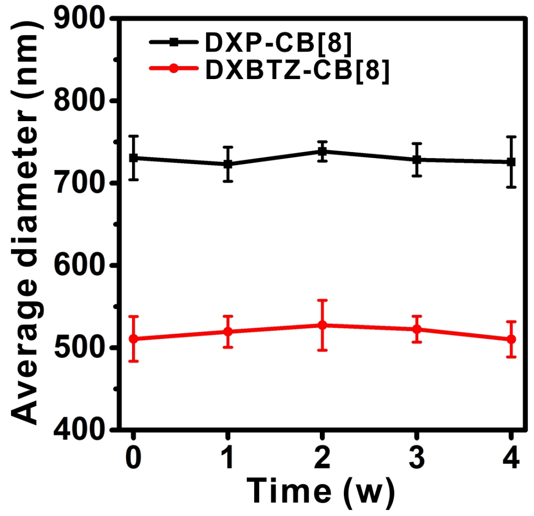

Supplementary Figure 24. Storage stability of the binary complexes DXP-CB[8] and DXBTZ-CB[8]. Average diameters of DXP-CB[8] and DXBTZ-CB[8] stored in water at room temperature for different times (n = 3 independent experiments). All data with error bars represent mean ± standard deviation (SD).

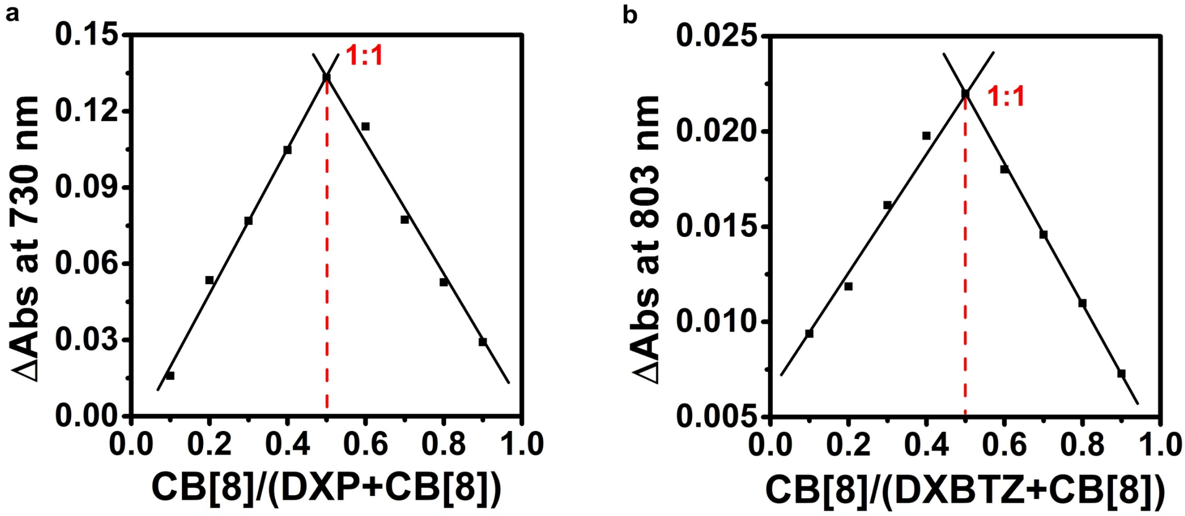

Supplementary Figure 25. Job plots for the complexation between dixanthene derivatives and CB[8]. (a) Job plot of DXP and CB[8] obtained by recording the absorbance at 730 nm in H_2O at 25 °C. (b) Job plot of DXBTZ and CB[8] obtained by recording the absorbance at 803 nm in H_2O containing 10% DMSO at 25 °C. The total concentration of dixanthene derivatives and CB[8] was fixed 10 μM.

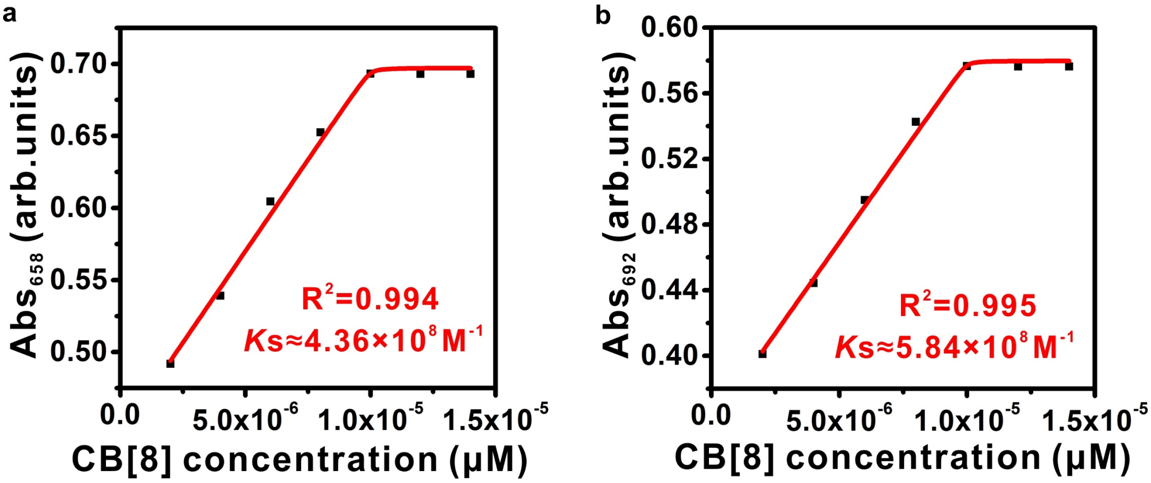

Supplementary Figure 26. Determination of the association constants (K_s) between dixanthene derivatives and CB[8]. The nonlinear least-squares analysis[4-6] of the absorbance of (a) DXP at 658 nm and (b) DXBTZ at 692 nm with varied concentrations of CB[8] for calculation of the association constants.

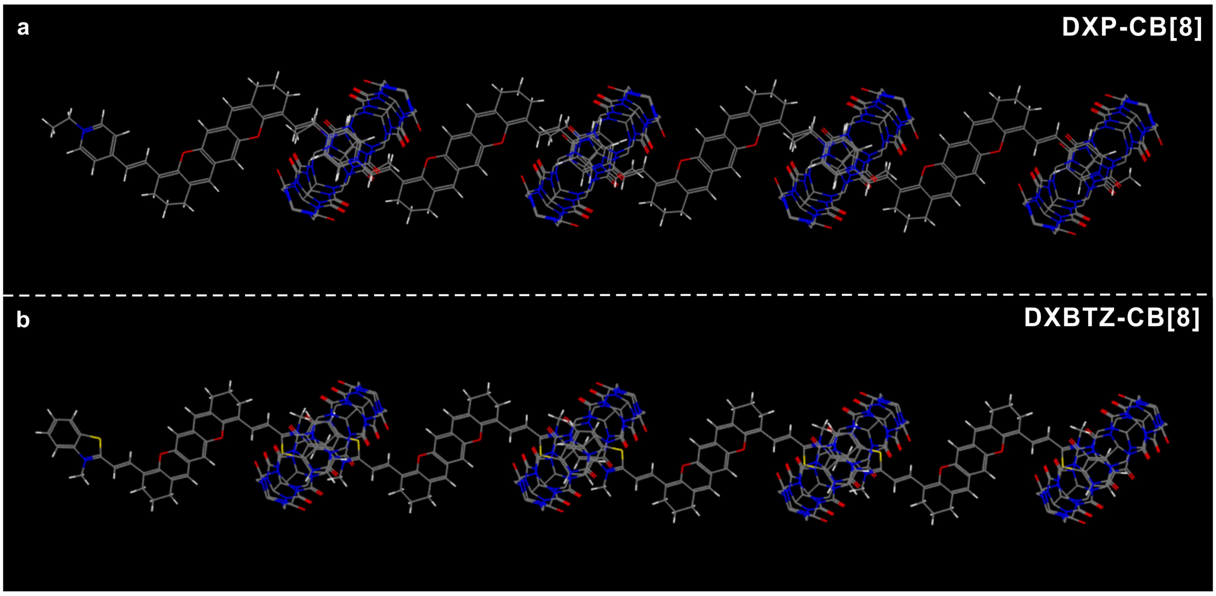

Supplementary Figure 27. Simulation for the sled binding mode of complexation between dixanthene derivatives and CB[8]. (a) DXP-CB[8] complexes. (b) DXBTZ-CB[8] complexes.

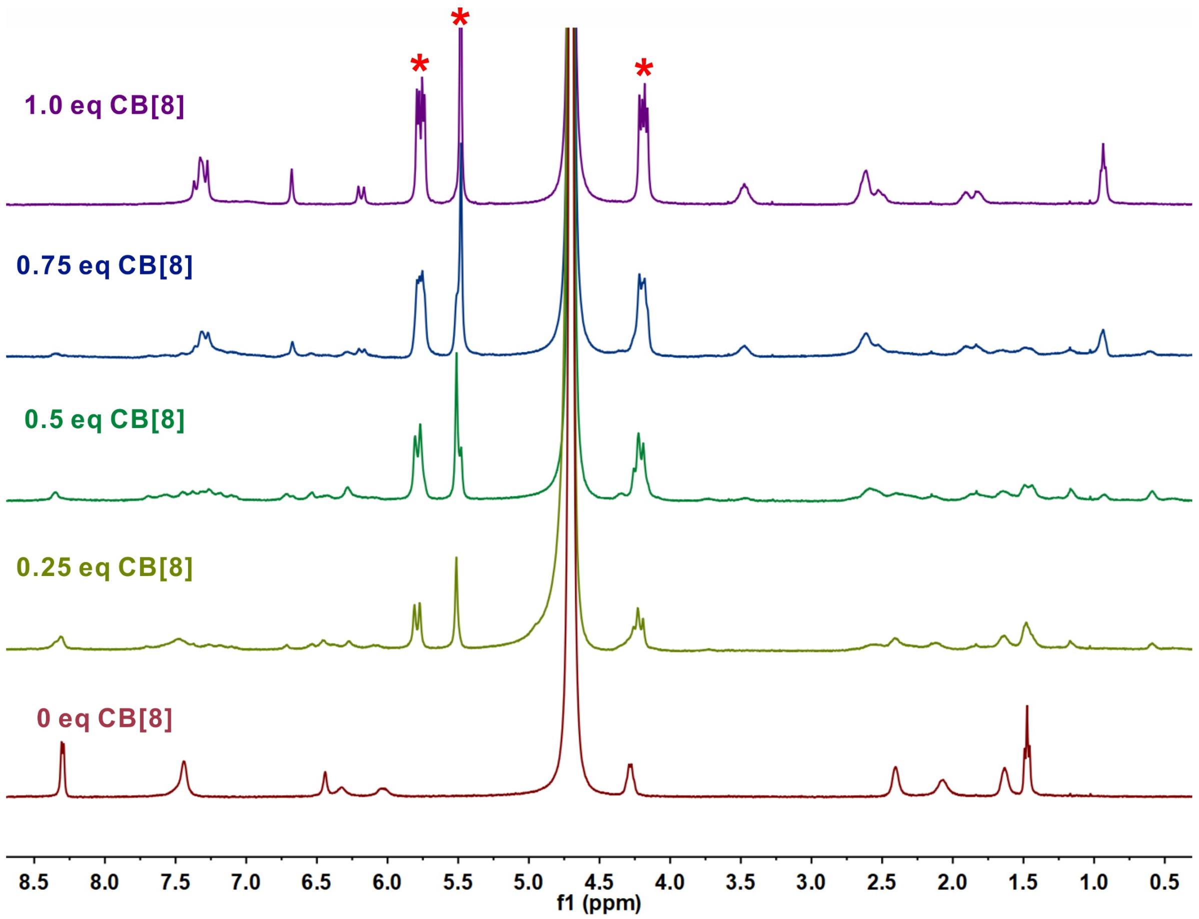

Supplementary Figure 28. NMR characterization of the complexation between DXP and CB[8]. ^{1}H NMR (400 MHz) spectral changes of DXP upon addition of varied concentrations of CB[8] in D_2O at 25 °C ([DXP] = 1.0 mM, [CB[8]] = 0, 0.25, 0.5, 0.75, 1.0 mM). The symbol '*' indicates the proton peak of CB[8].

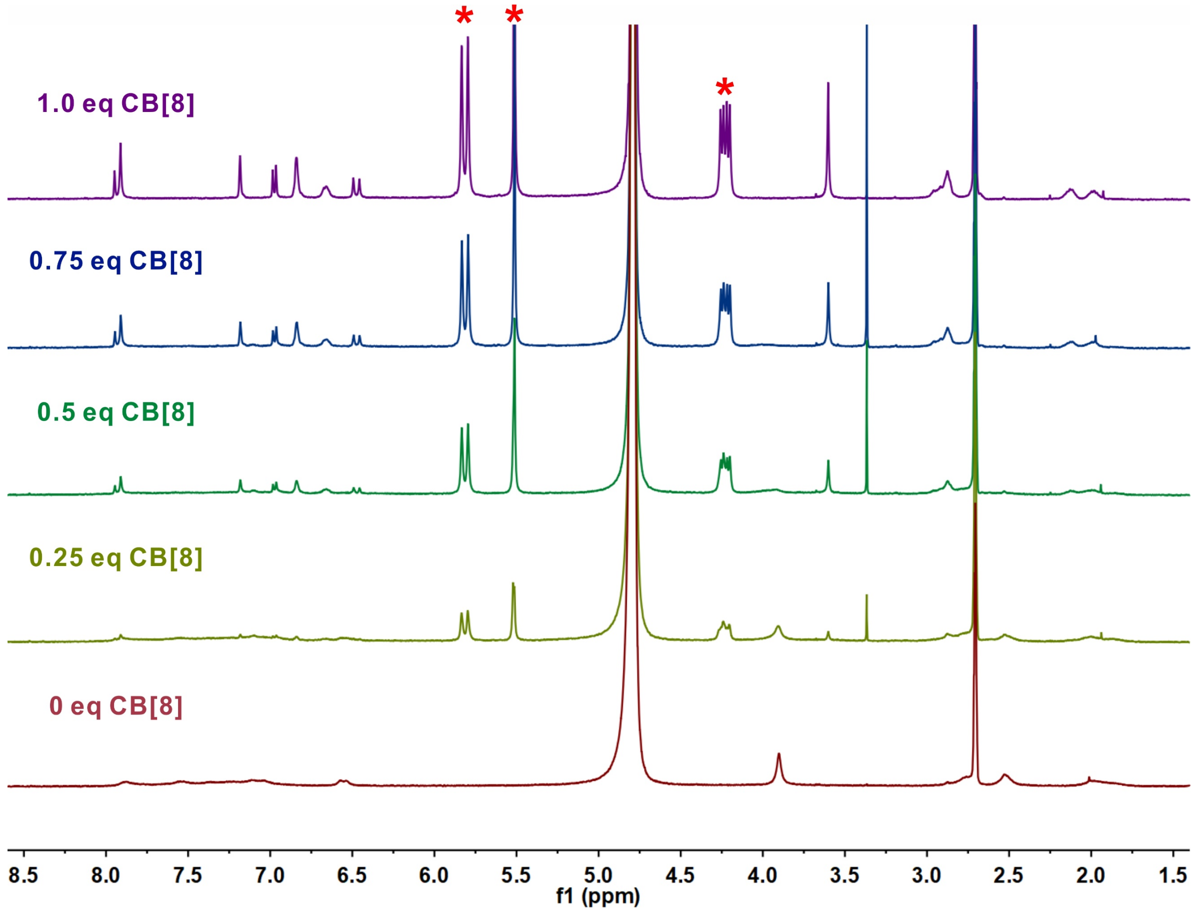

Supplementary Figure 29. NMR characterization of the complexation between DXBTZ and CB[8]. ^{1}H NMR (400 MHz) spectral changes of DXBTZ upon addition of varied concentrations of CB[8] in D_2O containing 10% DMSO-d_6 at 25 °C ([DXBTZ] = 1.0 mM, [CB[8]] = 0, 0.25, 0.5, 0.75, 1.0 mM). The symbol '*' indicates the proton peak of CB[8].

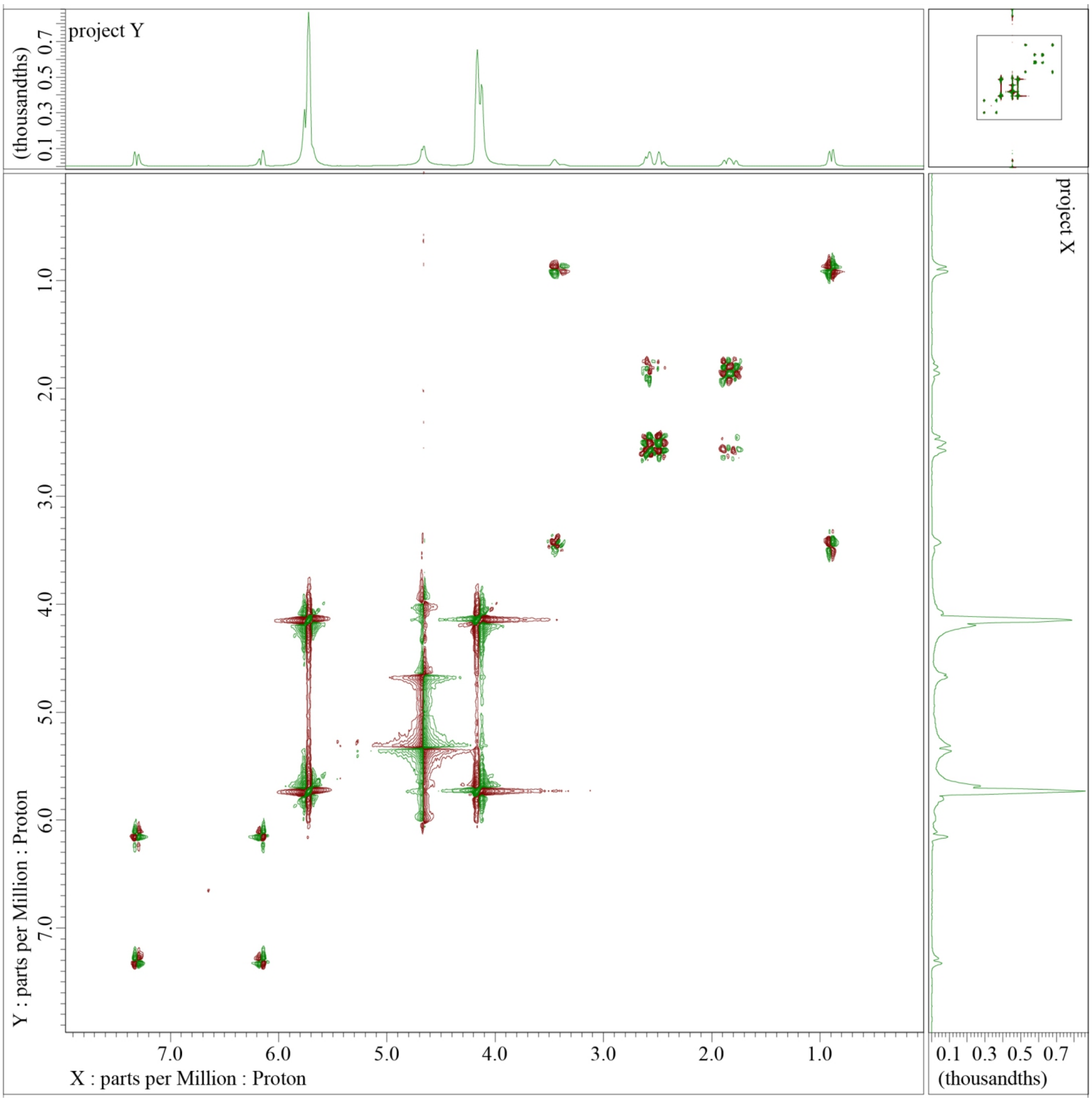

Supplementary Figure 30. DQF-COSY of DXP-CB[8]. 2D DQF-COSY spectra of DXP after addition of equimolar concentration of CB[8] in D_2O at 25 °C ([DXP] = [CB[8]] = 1.0 mM).

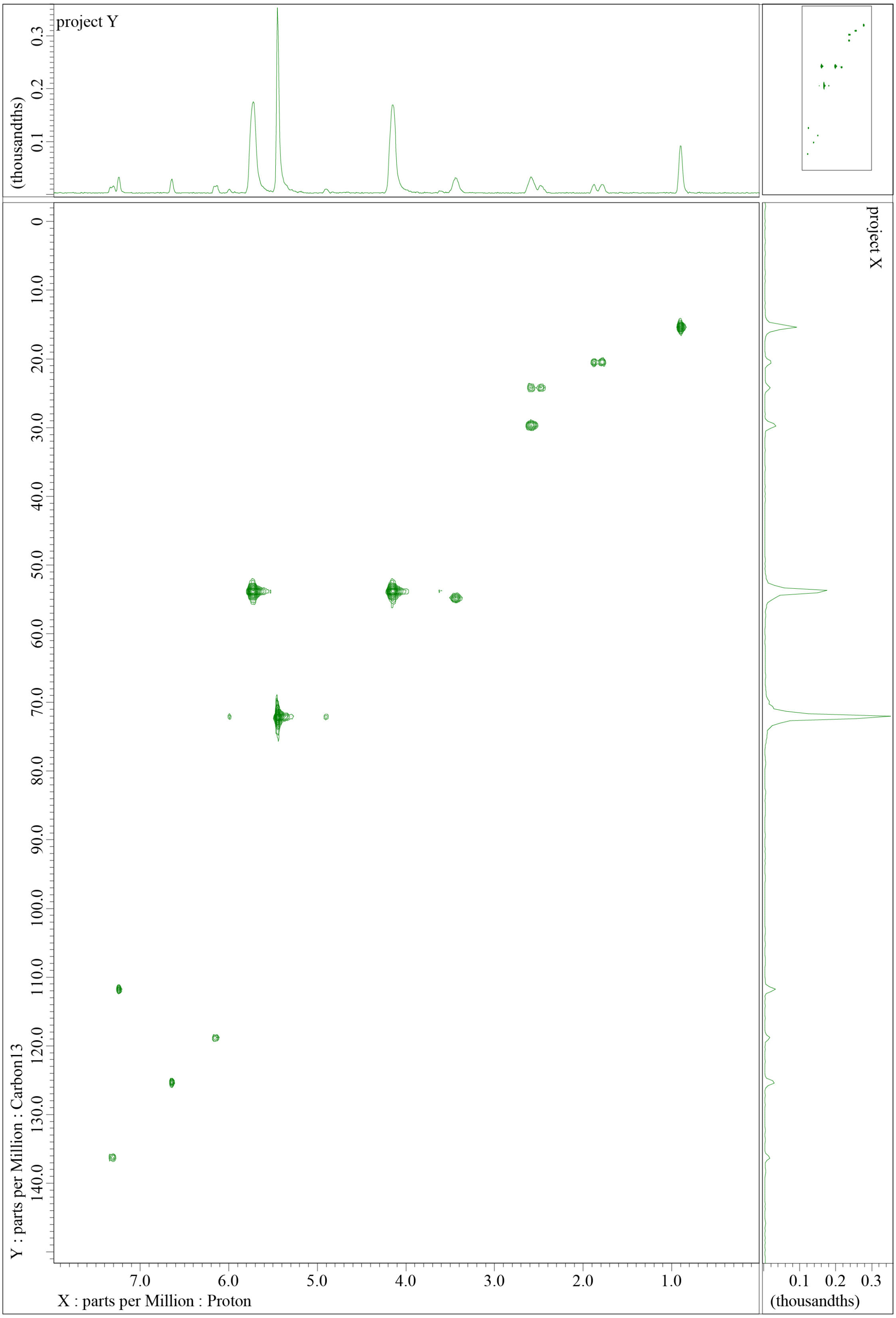

Supplementary Figure 31. HSQC of DXP-CB[8]. 2D HSQC spectra of DXP after addition of equimolar concentration of CB[8] in D_2O at 25 °C ([DXP] = [CB[8]] = 1.0 mM).

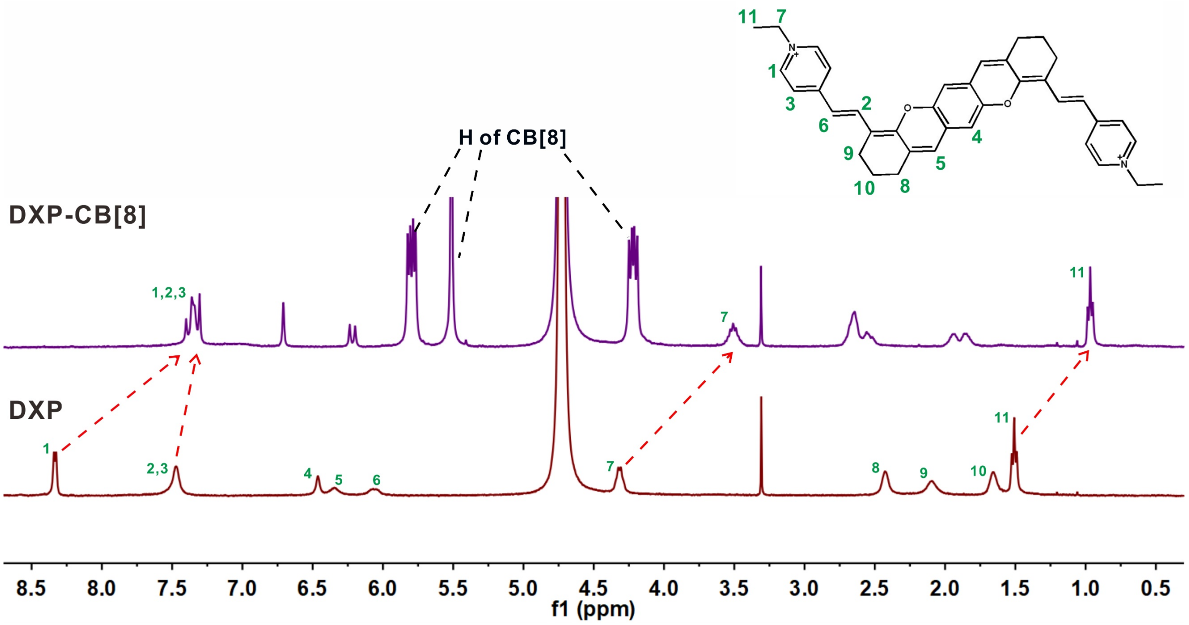

Supplementary Figure 32. Proton peak assignments for DXP and DXP-CB[8]. ^{1}H NMR spectra of DXP before (bottom) and after (top) addition of equimolar concentration of CB[8] in D_2O at 25 °C ([DXP] = [CB[8]] = 1.0 mM). A trace amount of methanol was added as an internal standard.

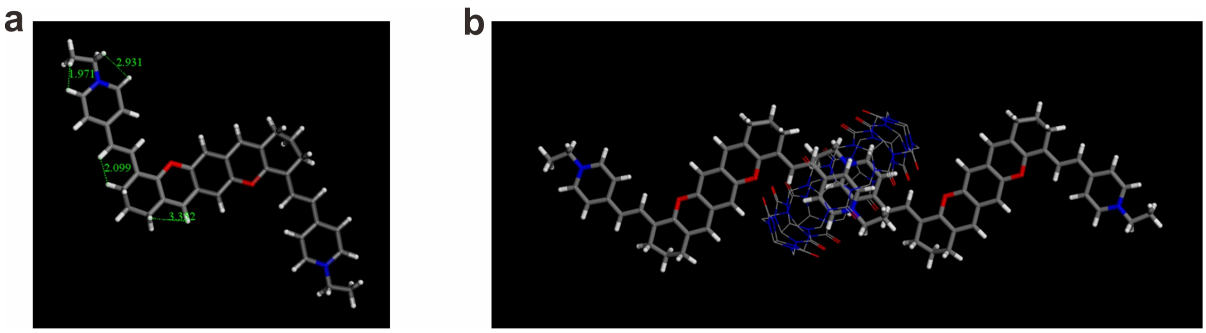

Supplementary Figure 33. Spacing calculations for DXP and DXP-CB[8]. (a) DXP and (b) DXP-CB[8].

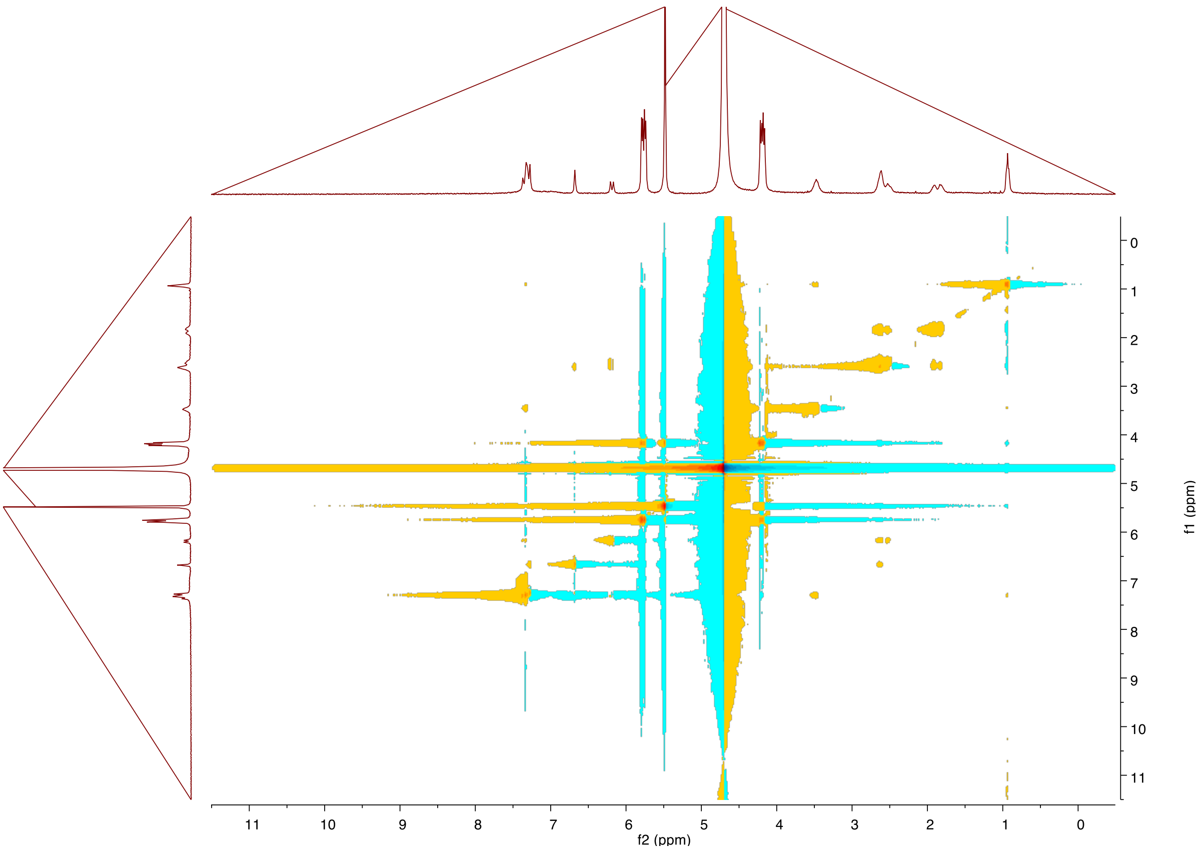

Supplementary Figure 34. NOESY of DXP-CB[8]. 2D NOESY spectra of DXP after addition of equimolar concentration of CB[8] in D_2O at 25 °C ([DXP] = [CB[8]] = 1.0 mM).

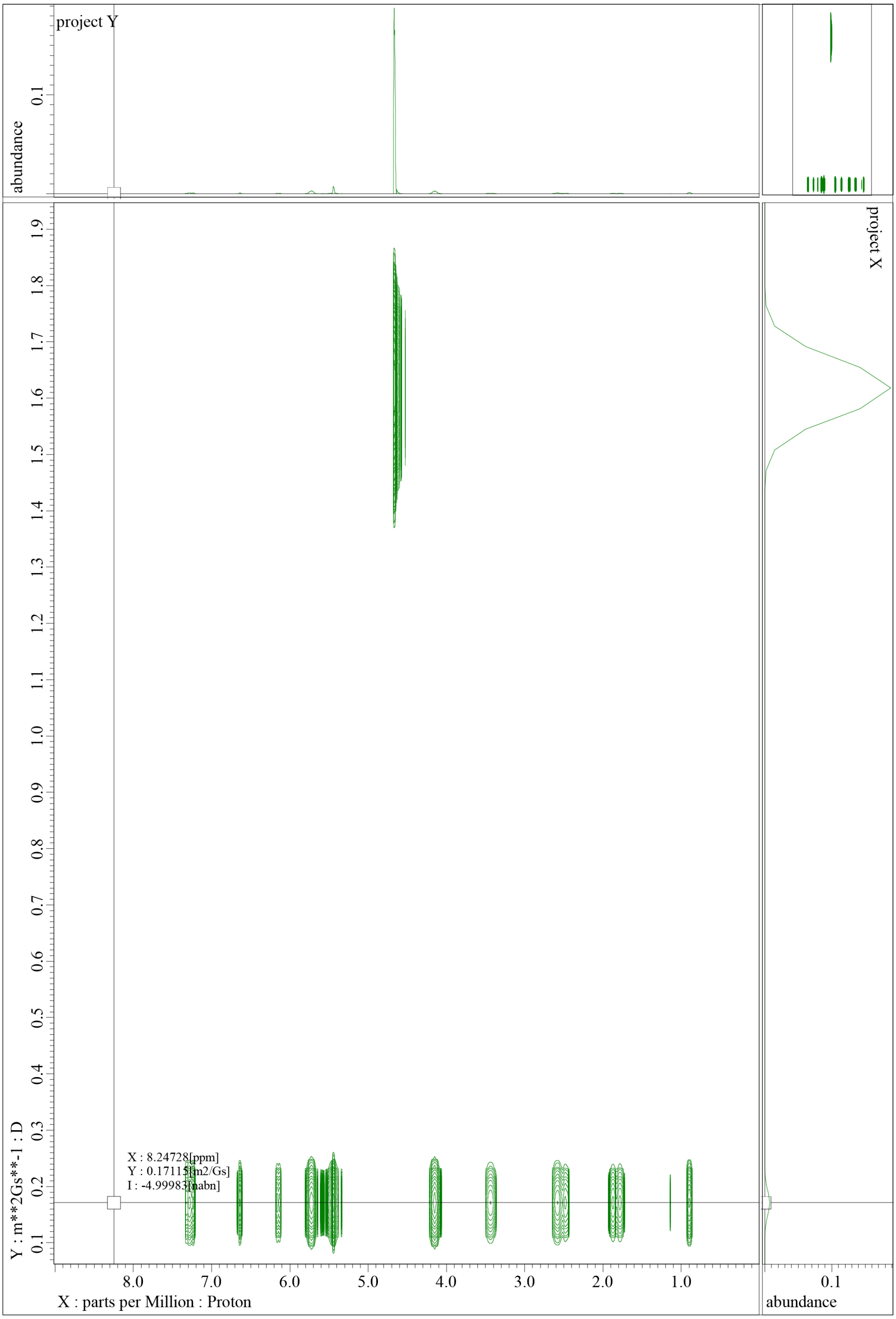

Supplementary Figure 35. DOSY of DXP-CB[8]. 2D DOSY spectra of DXP after addition of equimolar concentration of CB[8] in D_2O at 25 °C ([DXP] = [CB[8]] = 1.0 mM). The diffusion coefficient of DXP-CB[8] was determined as 1.7115×10^{-10} $m^2 \cdot s^{-1}$.

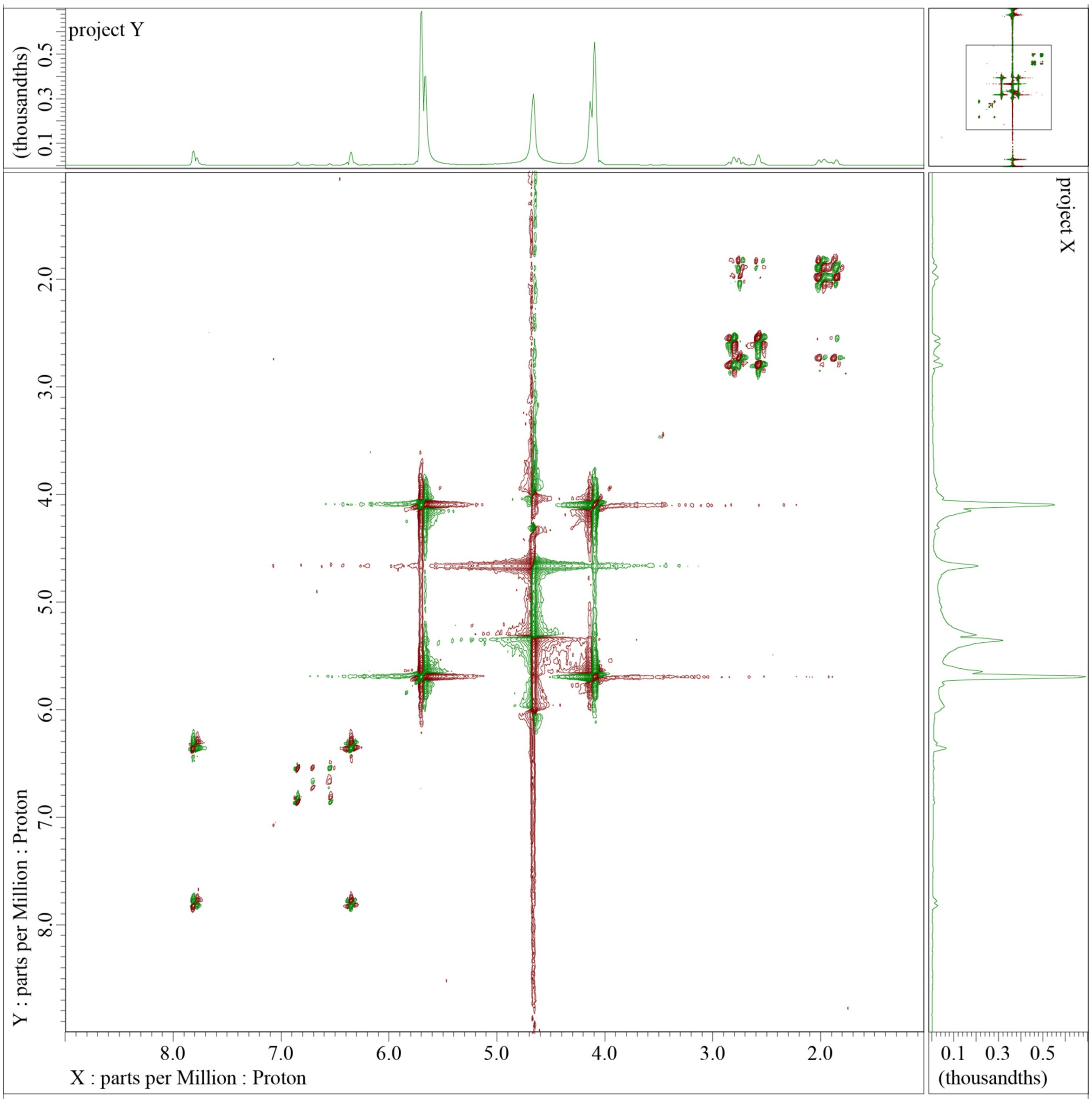

Supplementary Figure 36. DQF-COSY of DXBTZ-CB[8]. 2D DQF-COSY spectra of DXBTZ after addition of equimolar concentration of CB[8] in D_2O containing 10% DMSO-d_6 at 25 °C ([DXBTZ] = [CB[8]] = 1.0 mM).

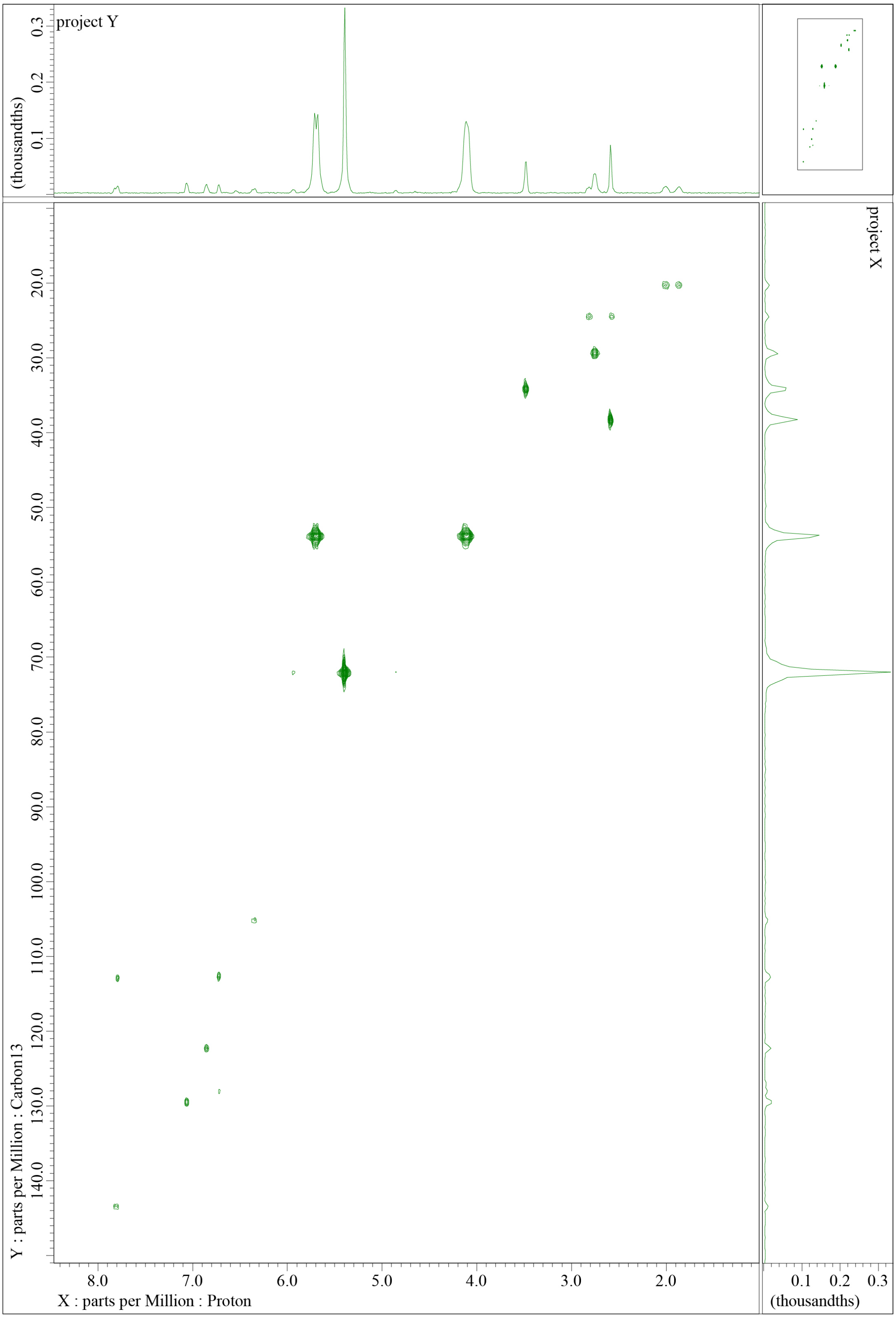

Supplementary Figure 37. HSQC of DXBTZ-CB[8]. 2D HSQC spectra of DXBTZ after addition of equimolar concentration of CB[8] in D_2O containing 10% DMSO-d_6 at 25 °C ([DXBTZ] = [CB[8]] = 1.0 mM).

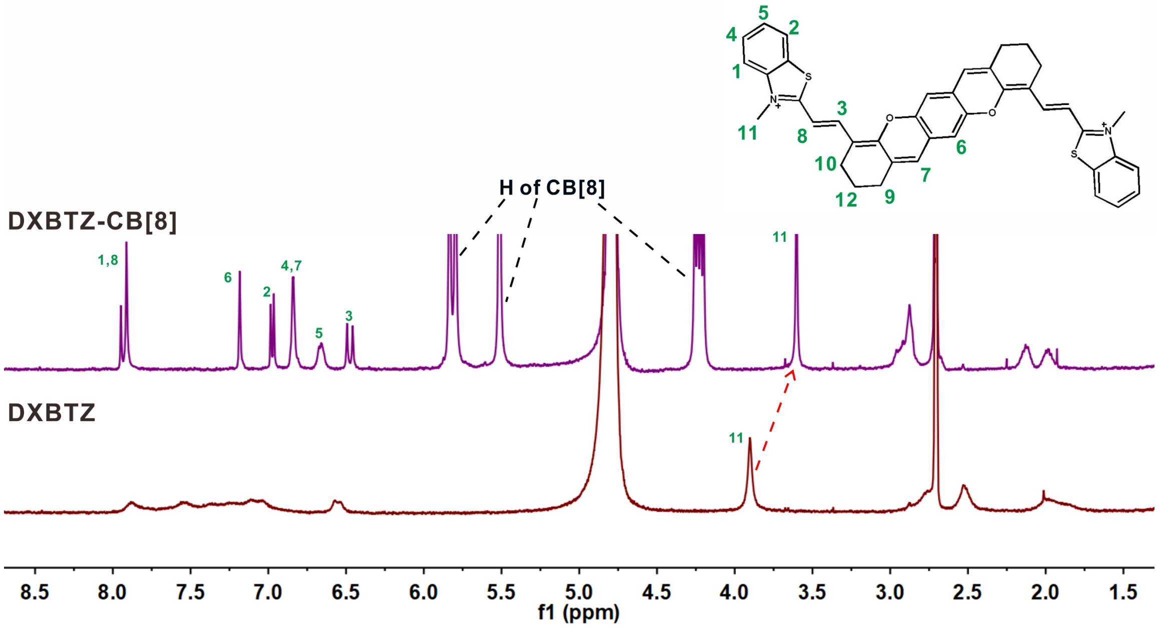

Supplementary Figure 38. Proton peak assignments for DXBTZ and DXBTZ-CB[8]. ^{1}H NMR spectra of DXBTZ before (bottom) and after (top) addition of equimolar concentration of CB[8] in D_2O containing 10% DMSO-d_6 at 25 °C ([DXBTZ] = [CB[8]] = 1.0 mM).

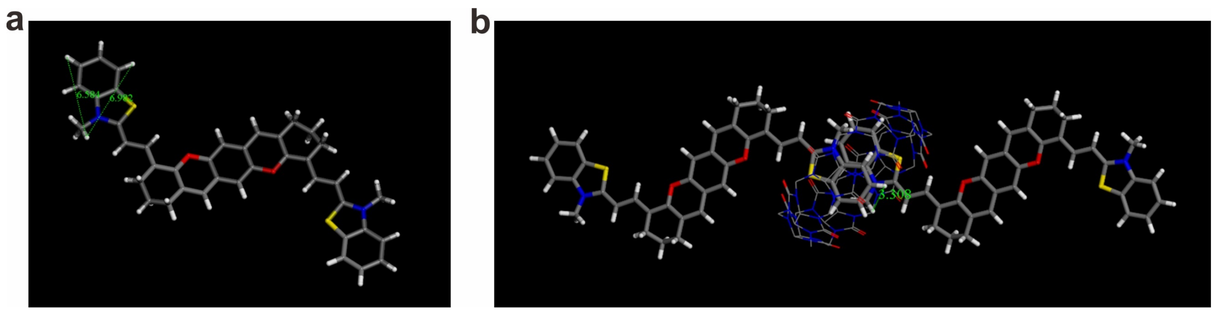

Supplementary Figure 39. Spacing calculations for DXBTZ and DXBTZ-CB[8]. (a) DXBTZ and (b) DXBTZ-CB[8].

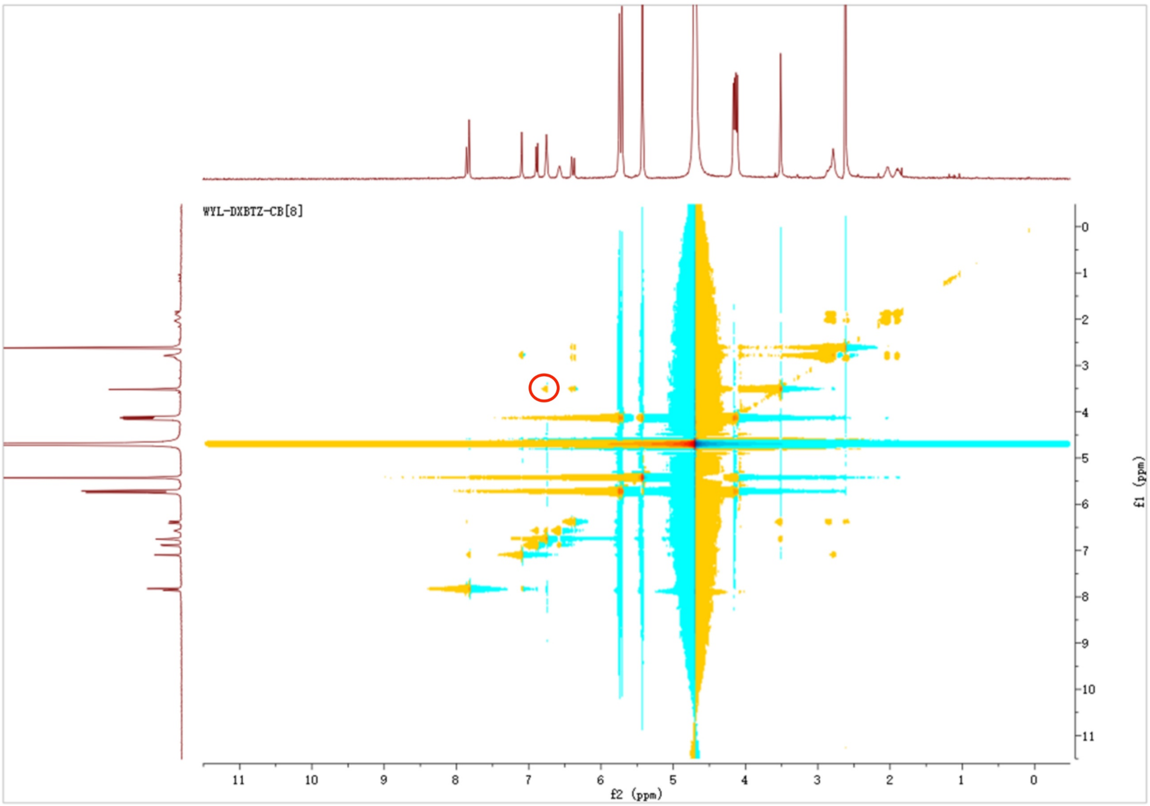

Supplementary Figure 40. NOESY of DXBTZ-CB[8]. 2D NOESY spectra of DXBTZ after addition of equimolar concentration of CB[8] in D_2O containing 10% DMSO-d_6 at 25 °C ([DXBTZ] = [CB[8]] = 1.0 mM). The red circle line indicates the cross peak between the aromatic Proton 4 and methyl group Proton 11.

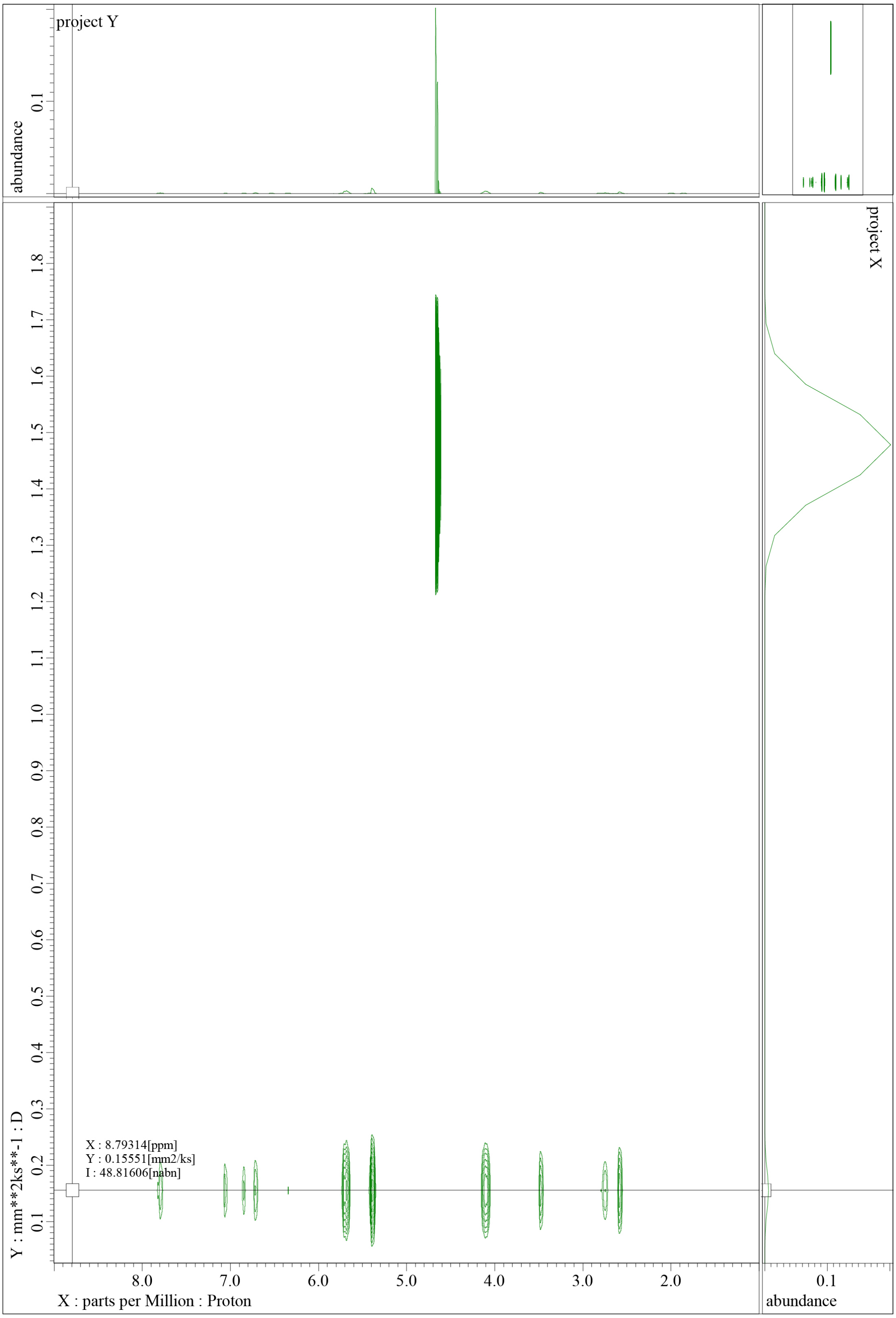

Supplementary Figure 41. DOSY of DXBTZ-CB[8]. 2D DOSY spectra of DXBTZ after addition of equimolar concentration of CB[8] in D_2O containing 10% DMSO-d_6 at 25 °C ([DXBTZ] = [CB[8]] = 1.0 mM). The diffusion coefficient of DXP-CB[8] was determined as 1.5115×10^{-10} $m^2 \cdot s^{-1}$.

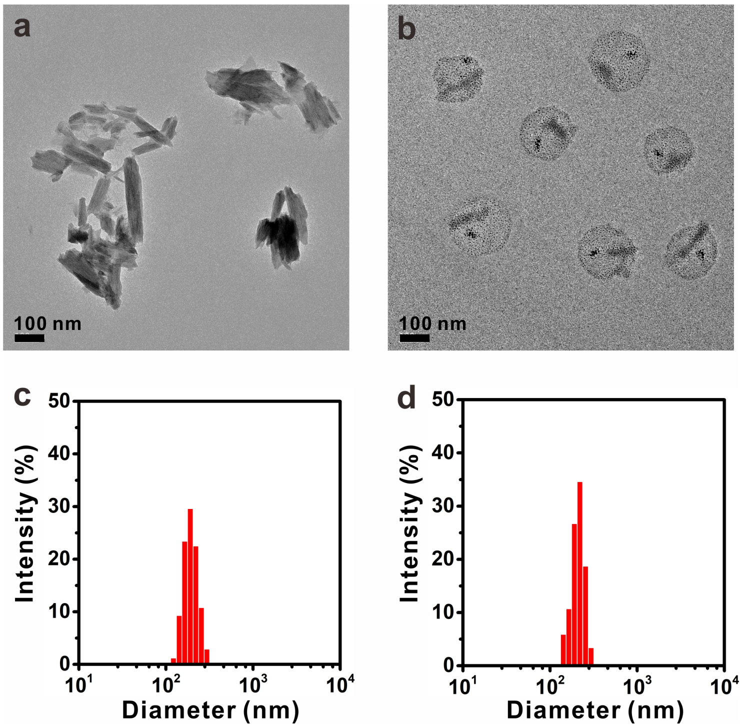

Supplementary Figure 42. Co-assembly process of DXBTZ-CB[8] and CSA. Representative transmission electron microscopic images and hydrodynamic diameter distribution for the sample of DXBTZ-CB[8] complexes (10 μM) at 2 min (a and c) or 5 min (b and d) after the addition of CSA (8 μg mL^{-1}). Scale bar: 100 nm.

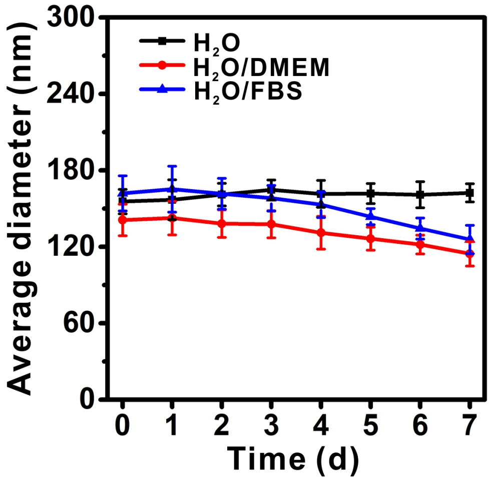

Supplementary Figure 43. Stability of the supramolecular nanoagent DXBTZ-CB[8]/CSA. Average diameters of the nanoagent DXBTZ-CB[8]/CSA stored in pure water, water containing 10% DMEM or water containing 10% FBS for different times (n = 3 independent experiments). All data with error bars represent mean ± standard deviation (SD).

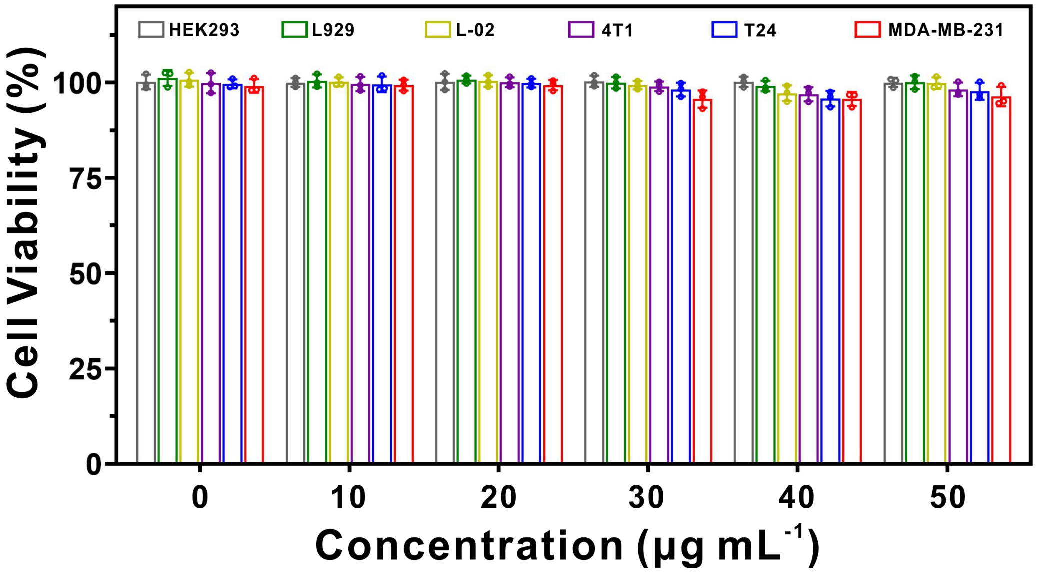

Supplementary Figure 44. Cell viabilities. Viabilities of HEK293, L929, L-02, 4T1, T24 and MDA-MB-231 cells upon 24 hours of incubation with the nanoagent DXBTZ-CB[8]/CSA at different concentrations (0, 10, 20, 30, 40 and 50 μg mL^{-1}). Data were represented as mean ± SD from three independent experiments.

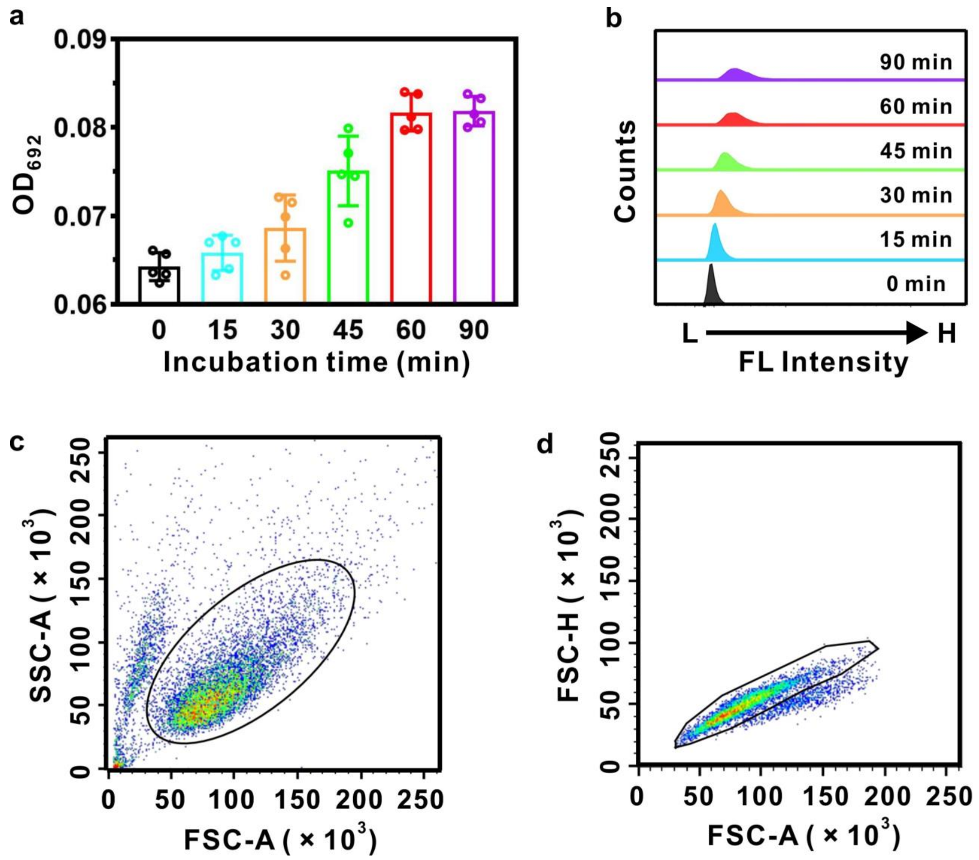

Supplementary Figure 45. Cellular uptake. (a) Optical density at 692 nm (n = 3 independent experiments) and (b) flow cytometry profiles for MDA-MB-231 cells incubated with the nanoagent DXBTZ-CB[8]/CSA (30 μg mL^{-1}) for 0, 15, 30, 45, 60 or 90 min. Gating strategy for (b) was determined by (c) FSC-A versus SSC-A plot and then (d) FSC-A versus FSC-H plot. All data with error bars represent mean ± standard deviation (SD).

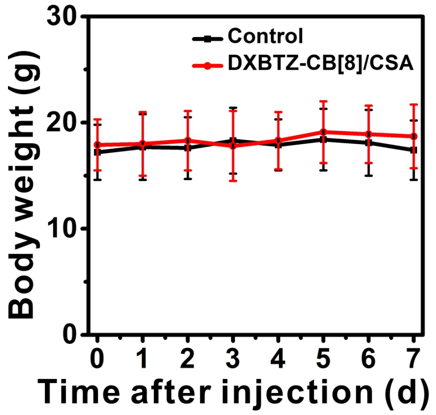

Supplementary Figure 46. Body weight measurements. Time-dependent changes in mouse body weight for the control mice, and the mice after intravenous injection of the nanoagent DXBTZ-CB[8]/CSA (62.2 mg kg^{-1}) (n = 5 animals per group). All data with error bars represent mean ± standard deviation (SD).

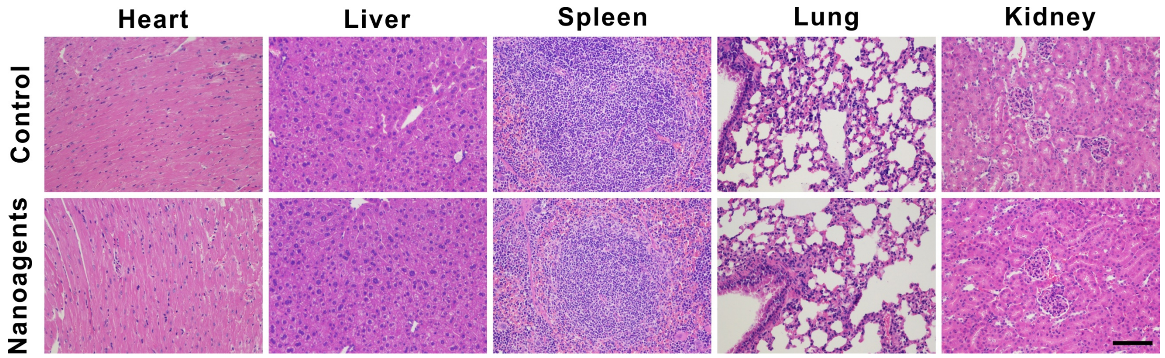

Supplementary Figure 47. H&E staining analysis. Representative histological sections for main organs of the control mice, and the mice 7 days after administration with the nanoagent DXBTZ-CB[8]/CSA (62.2 mg kg^{-1}). Scale bar: 100 μm.

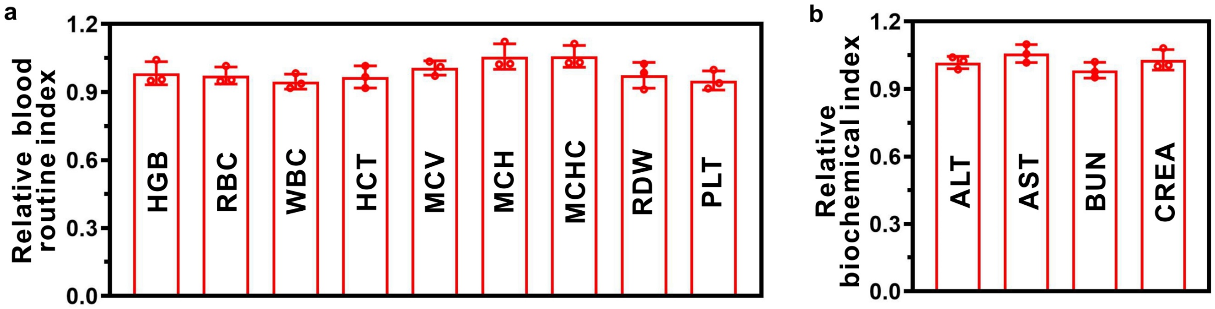

Supplementary Figure 48. Determination of blood routine indicators and serum biochemical parameters. (a) Relative blood routine indexes and (b) relative biochemical indexes of mice 7 days after injection of the nanoagent DXBTZ-CB[8]/CSA (62.2 mg kg^{-1}) (n = 3 animals per group). HGB: hemoglobin, RBC: red blood cells, WBC: white blood cells, HCT: hematocrit, MCV: mean corpuscular volume, MCH: mean corpuscular hemoglobin, MCHC: mean corpuscular hemoglobin concentration, RDW: red cell distribution width, PLT: platelets, ALT: alanine transaminase, AST: aspartate aminotransferase, BUN: blood urea nitrogen, and CREA: creatinine. **Note:** These relative indexes were used to reflect the differences in blood routine indicators and biochemical parameters between the mice injected with DXBTZ-CB[8]/CSA and the control mice. They were calculated according to the equations (1) or (2):

Relative blood routine indexes = (Mean blood routine value)$_{\text{DXBTZ-CB[8]/CSA}}$ / (Mean blood routine value)$_{\text{control}}$ (1)

Relative biochemical indexes = (Mean biochemical value)$_{\text{DXBTZ-CB[8]/CSA}}$ / (Mean biochemical value)$_{\text{control}}$ (2)

All data with error bars represent mean ± standard deviation (SD).

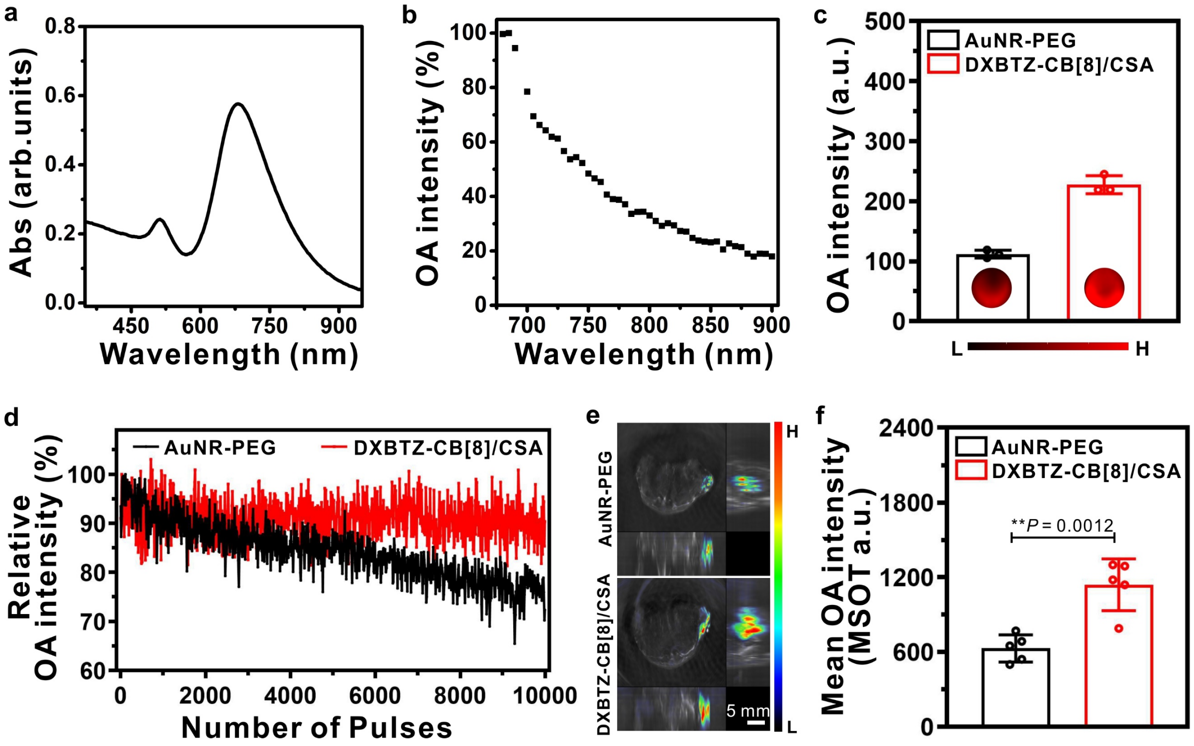

Supplementary Figure 49. Comparison with commercially available PEG$_{5000}$-functionalized gold nanorods (AuNR-PEG). (a) Absorption spectra and (b) optoacoustic spectra of AuNR-PEG. (c) Optoacoustic intensity at 692 nm of AuNR-PEG and DXBTZ-CB[8]/CSA at the same mass concentration (30 μg mL^{-1}) in phantom (n = 3 independent experiments). (d) Relative OA intensity (%) versus number of laser pulses for AuNR-PEG and DXBTZ DXBTZ-CB[8]/CSA at the same mass concentration (30 μg mL^{-1}) in phantom. (e) Representative z-stack orthogonal MIP MSOT images and (f) mean OA intensity of the tumor regions for mice intratumorally injected with the same concentration (15.6 mg kg^{-1}) of AuNR-PEG or DXBTZ-CB[8]/CSA (n = 5 animals per group). All data with error bars represent mean ± standard deviation (SD). Statistical significance was determined by two-tailed *t* test. $^{**}P < 0.01$.

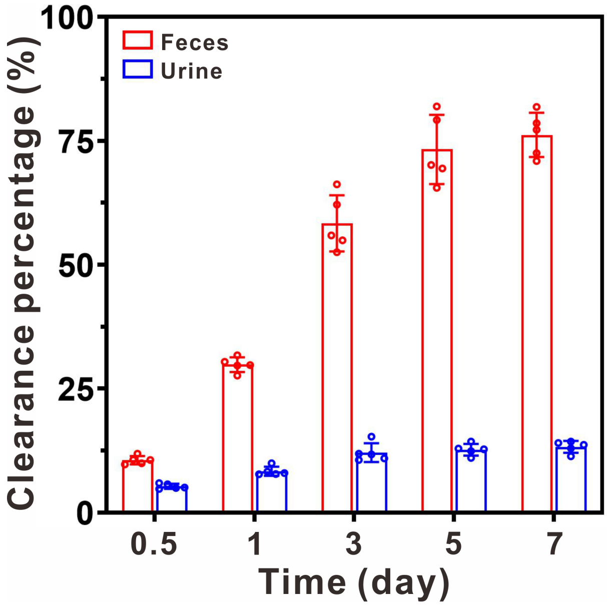

Supplementary Figure 50. Clearance behavior. Cumulative clearance percentage of the nanoagent DXBTZ-CB[8]/CSA in subcutaneous tumor-bearing mice determined by measuring optoacoustic intensity of feces and urine solution at different time points after the intravenous administration (n = 5 animals per group). All data with error bars represent mean ± standard deviation (SD).

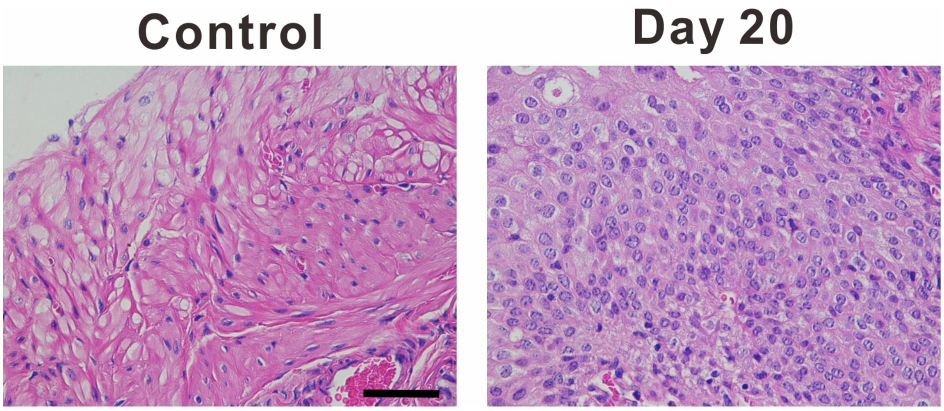

Supplementary Figure 51. H&E staining analysis. Magnified H&E images of the areas in the black dotted box in Fig. 5m. Scale bar: 50 μm.

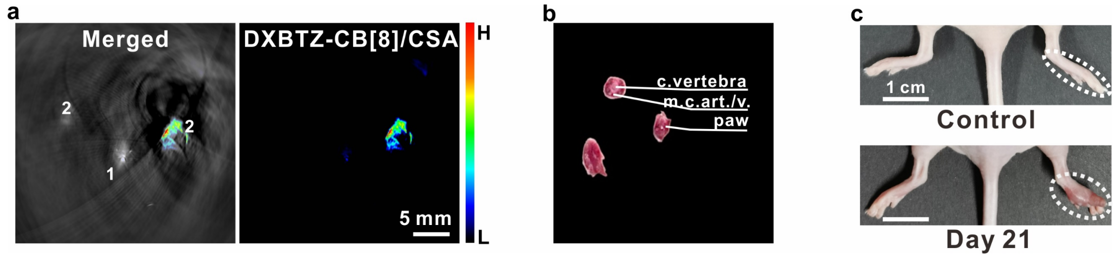

Supplementary Figure 52. MSOT imaging of the primary tumor site in mice with lymphatic metastasis. (a) Typical cross-sectional MSOT images corresponding to the paw location of mice 21 days post intra-footpad inoculation of 4T1 cells. Left panel: overlay of DXBTZ-CB[8]/CSA optoacoustic signal onto the grayscale image with anatomical information. Right panel: spectrally unmixed optoacoustic signal of DXBTZ-CB[8]/CSA. Organ tags: 1: tail bone; 2: paw. Scale bar: 5 mm. (b) Cryosection image of a female mouse corresponding to the cross-section location in (a). (c) Pictures for hind paws and legs of the control mouse and the mouse 21 days post intra-footpad inoculation of 4T1 cells.

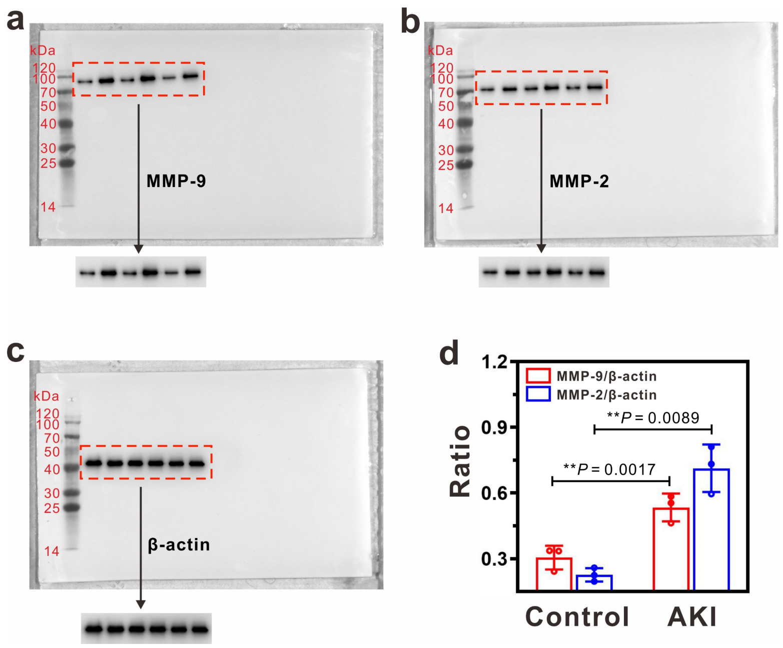

Supplementary Figure 53. Uncropped western blots of MMP-9, MMP-2, and β-actin. Original whole WB membrane of (a) MMP-9, (b) MMP-2, and (c) β-actin expressed in the kidneys of control and AKI mice. (d) Ratios of MMP-9/β-actin and MMP-2/β-actin in the kidneys of control and AKI mice (n = 3 animals per group). All data with error bars represent mean ± standard deviation (SD). Statistical significance was determined by two-tailed *t* test. $^{**}P < 0.01$.

Supplementary Table 1. Optimized geometric structure of DXP in DMSO at the ground state.

Atom	X	Y	Z
C	-4.32796283	-4.36060227	0.30945718
C	-3.16189061	-5.14441417	0.89831559
C	-1.88148803	-4.80359458	0.14345115
C	-1.62062513	-3.32287616	0.16854755
C	-2.75228366	-2.43369957	0.18205099
C	-4.06426616	-2.86516108	0.22105927
C	-0.36322587	-2.81079971	0.16363156
C	-0.13260192	-1.40022444	0.15567427
C	-1.26063648	-0.55751488	0.1538465
O	-2.52176655	-1.08499118	0.15735839
C	1.13984085	-0.81685898	0.15448499
C	1.26056847	0.55736825	0.15376807
C	0.13253465	1.40007531	0.15568391
C	-1.13990837	0.81671409	0.15424042
C	-5.21571935	-2.0359767	0.17227498
C	-5.35710196	-0.6830581	0.01455037
C	-6.61727545	-0.0126193	-0.02915246
C	-6.66259488	1.38532663	-0.26220411
C	-7.84893249	2.06053451	-0.3118268
N	-9.03181821	1.42231157	-0.1317264
C	-9.03546356	0.08321861	0.09426739
C	-7.87850344	-0.63771797	0.15355933
C	-10.31176944	2.15981052	-0.26690538
C	-10.8296677	2.14296008	-1.69787184
H	-5.22477446	-4.52096373	0.91087761
H	-4.55698475	-4.74565221	-0.69103888
H	-3.36058603	-6.2148633	0.84136704
H	-3.04255784	-4.89169086	1.95541637
H	-1.02017233	-5.33135363	0.55450964
H	-1.9813734	-5.12372912	-0.90000629
H	0.48926537	-3.47898276	0.16116263
H	2.02421127	-1.43916564	0.16146082
H	-2.02427558	1.43902414	0.16121977
H	-6.13462093	-2.60739012	0.25915495
H	-4.48387292	-0.06118962	-0.10095918
H	-5.74590351	1.93945519	-0.407005
H	-7.90522647	3.12426662	-0.49214036
H	-10.00623165	-0.37121671	0.23004238
H	-7.9582903	-1.69565415	0.35135117
H	-11.02006229	1.69841949	0.41962976

H	-10.13245276	3.17591809	0.08104928
H	-11.76819477	2.69530693	-1.75000065
H	-10.12064046	2.61295218	-2.38061469
H	-11.01692887	1.1241161	-2.03924992
C	4.32788857	4.36045732	0.30939814
C	3.16178972	5.14429771	0.89816827
C	1.88142211	4.80344495	0.1432607
C	1.62055603	3.32272689	0.1684231
C	2.75221345	2.4335532	0.18199386
C	4.06419465	2.86501561	0.22104707
C	0.36315823	2.81065038	0.16348808
O	2.52169851	1.08484327	0.15735674
C	5.21564117	2.03581131	0.172397
C	5.35701666	0.6829419	0.01424925
C	6.6171829	0.01247574	-0.029286
C	6.6626151	-1.38518982	-0.26395928
C	7.84897339	-2.06037486	-0.31354629
N	9.03174349	-1.42241093	-0.13182042
C	9.03527326	-0.08359602	0.09584489
C	7.87829299	0.63730024	0.15518829
C	10.31178082	-2.15977273	-0.26699461
C	10.8307804	-2.14100211	-1.69753432
H	5.22467867	4.52084513	0.91084483
H	4.55695514	4.74546484	-0.69110393
H	3.3604864	6.21474413	0.84117721
H	3.04240828	4.89162292	1.95527523
H	1.02009019	5.33122662	0.55425568
H	1.98135518	5.12353191	-0.9002069
H	-0.48933194	3.47883409	0.16096649
H	6.13454235	2.60717299	0.25963162
H	4.48380809	0.06117692	-0.1019832
H	5.74601422	-1.93910595	-0.4101403
H	7.90536527	-3.12388514	-0.49513783
H	10.00595801	0.37061939	0.23294737
H	7.95794157	1.69496513	0.35448216
H	11.01955417	-1.69934144	0.42072111
H	10.13215033	-3.17634276	0.07944401
H	11.76940765	-2.69317747	-1.74966841
H	10.1223233	-2.6101671	-2.38143659
H	11.01819657	-1.12169111	-2.03743478

Supplementary Table 2. Optimized geometric structure of DXBTZ in DMSO at the ground state.

Atom	X	Y	Z
C	4.7842629	3.86078974	0.29663086
C	3.70059933	4.7804436	0.85491892
C	2.38910405	4.57444723	0.10320772
C	1.9575849	3.13533146	0.16055249
C	2.98310939	2.13085896	0.18581237
C	4.34074284	2.4170988	0.2020791
C	0.6523792	2.75809574	0.16405702
C	0.27590182	1.37893615	0.16883729
C	1.31020176	0.42176531	0.16988254
O	2.61716455	0.81722836	0.18021079
C	-1.05079771	0.93286483	0.16796734
C	-1.31022126	-0.42181187	0.16983683
C	-0.27591572	-1.37898015	0.16872294
C	1.05078425	-0.93291081	0.16831785
C	5.28534529	1.37598443	0.10986683
C	6.64933561	1.54225065	0.07839398
C	10.6850462	-3.09870116	-0.59762729
C	11.52604385	-1.98873452	-0.48046926
C	11.0142679	-0.71290718	-0.29552519
C	9.63109271	-0.56350839	-0.22704233
C	8.79419635	-1.67778361	-0.35393985
C	9.3060394	-2.95438629	-0.53726914
N	8.90702742	0.62142241	-0.0367272
C	7.56518627	0.47997359	-0.04907907
S	7.10227291	-1.19532467	-0.26145317
C	9.56362357	1.91875404	0.13929297
H	5.66676056	3.92170055	0.93638692
H	5.09506369	4.21137283	-0.69425556
H	4.01888861	5.82020413	0.77889696
H	3.5491991	4.56606723	1.91615832
H	1.59721769	5.20957768	0.50146607
H	2.52367074	4.85679406	-0.94723692
H	-0.12601345	3.51128639	0.15072108
H	-1.86832657	1.64085629	0.16851537
H	1.8683026	-1.64091242	0.16888622
H	4.88835192	0.36923213	0.04676671
H	7.06462555	2.53708238	0.13069131
H	11.1118911	-4.08188675	-0.7409965
H	12.59758195	-2.12289145	-0.53740588
H	11.68199122	0.13267335	-0.21882528

H	8.65211369	-3.81026566	-0.63287875
H	9.04229508	2.49343031	0.90020597
H	10.57985704	1.75361007	0.47979335
H	9.58021476	2.46995558	-0.80126115
C	-4.78427828	-3.86082849	0.29657484
C	-3.70062471	-4.78046325	0.85491896
C	-2.38911856	-4.57449299	0.103206
C	-1.95760751	-3.13538105	0.16048958
C	-2.98313187	-2.13089792	0.1856772
C	-4.34076431	-2.41713769	0.20200285
C	-0.65240079	-2.75813945	0.16407096
O	-2.61718307	-0.8172852	0.17991347
C	-5.28535889	-1.37601231	0.10981924
C	-6.649351	-1.54225826	0.07830941
C	-10.68499381	3.09877037	-0.59759227
C	-11.52601104	1.98882921	-0.48032987
C	-11.01425356	0.71299596	-0.29537521
C	-9.63107769	0.56356684	-0.22698607
C	-8.79416212	1.67781471	-0.35398923
C	-9.30598627	2.95442364	-0.53732988
N	-8.90702753	-0.62137621	-0.03667735
C	-7.56518505	-0.47996029	-0.0491331
S	-7.10224296	1.19531166	-0.26163328
C	-9.5636441	-1.91867863	0.13948819
H	-5.666794	-3.92172945	0.93630521
H	-5.09504431	-4.21144745	-0.69431016
H	-4.01891415	-5.8202263	0.77893555
H	-3.54923146	-4.56603967	1.91614936
H	-1.59723431	-5.20961358	0.50148351
H	-2.52369361	-4.85687907	-0.94722685
H	0.12598359	-3.51134108	0.15080226
H	-4.88835617	-0.36925872	0.04678691
H	-7.06466026	-2.53708532	0.13053497
H	-11.11182533	4.08196081	-0.74096828
H	-12.59754983	2.12301124	-0.53719463
H	-11.6819919	-0.1325656	-0.21859342
H	-8.65204553	3.8102823	-0.63302179
H	-9.04227057	-2.49330853	0.9004058
H	-10.57984894	-1.75348139	0.48004807
H	-9.58031932	-2.46995088	-0.80102303

Supplementary References

[1] Frisch, M. J. et al. *Gaussian 16, Rev. A.01* (2016). https://gaussian.com/

[2] Lu, T. & Chen, F. Multiwfn: a multifunctional wavefunction analyzer. *J. Comput. Chem.* **33**, 580–592 (2012).

[3] Humphrey, W., Dalke, A. & Schulten, K. VMD: visual molecular dynamics. *J. Mol. Graphics* **14**, 33-38 (1996).

[4] Hargrove, A. E., Zhong, Z., Sessler, J. L. & Anslyn, E. V. Algorithms for the determination of binding constants and enantiomeric excess in complex host: guest equilibria using optical measurements. *New J. Chem.* **34**, 348-354 (2010).

[5] Chen, W. et al. Molecular cursor caliper: a fluorescent sensor for dicarboxylate dianions. *J. Am. Chem. Soc.* **141**, 14798-14806 (2019).

[6] Geng, W. C. et al. Supramolecular bioimaging through signal amplification by combining indicator displacement assay with Förster resonance energy transfer. *Angew. Chem. Int. Ed.* **60**, 19614-19619 (2021).
