# Supplementary material for: Cucurbit[8]uril-based water-dispersible assemblies with enhanced optoacoustic performance for multispectral optoacoustic imaging
Source: Nat Commun. 2023 Jul 3;14:3918. doi: 10.1038/s41467-023-39610-2 (PMC10317952; doi:10.1038/s41467-023-39610-2)
Supplement: Supplementary file 2 — Reporting Summary [file 41467_2023_39610_MOESM2_ESM.pdf]

nature portfolio

Corresponding author(s): Yanli Zhao; Shuizhu Wu; Hongzhong Chen

Last updated by author(s): Jun 13, 2023

Reporting Summary

Nature Portfolio wishes to improve the reproducibility of the work that we publish. This form provides structure for consistency and transparency in reporting. For further information on Nature Portfolio policies, see our Editorial Policies and the Editorial Policy Checklist.

Statistics

For all statistical analyses, confirm that the following items are present in the figure legend, table legend, main text, or Methods section.

n/a	Confirmed	
☐	☒	The exact sample size (*n*) for each experimental group/condition, given as a discrete number and unit of measurement
☐	☒	A statement on whether measurements were taken from distinct samples or whether the same sample was measured repeatedly
☐	☒	The statistical test(s) used AND whether they are one- or two-sided<br>*Only common tests should be described solely by name; describe more complex techniques in the Methods section.*
☐	☒	A description of all covariates tested
☐	☒	A description of any assumptions or corrections, such as tests of normality and adjustment for multiple comparisons
☐	☒	A full description of the statistical parameters including central tendency (e.g. means) or other basic estimates (e.g. regression coefficient) AND variation (e.g. standard deviation) or associated estimates of uncertainty (e.g. confidence intervals)
☐	☒	For null hypothesis testing, the test statistic (e.g. *F*, *t*, *r*) with confidence intervals, effect sizes, degrees of freedom and *P* value noted<br>*Give P values as exact values whenever suitable.*
☒	☐	For Bayesian analysis, information on the choice of priors and Markov chain Monte Carlo settings
☒	☐	For hierarchical and complex designs, identification of the appropriate level for tests and full reporting of outcomes
☒	☐	Estimates of effect sizes (e.g. Cohen's *d*, Pearson's *r*), indicating how they were calculated

Our web collection on statistics for biologists contains articles on many of the points above.

Software and code

Policy information about availability of computer code

Data collection	Data of in vitro fluoresence imaging are collected with the Living Image 4.3 software of IVIS fluorescence imaging system. Data of in vitro test and in vivo animal optoacoustic imaging are collected with viewMSOT 3.8 of inVision128 multispectral optoacoustic tomographic (MSOT) imaging system (iThera Medical GmbH). Data of cell fluorescence imaging were collected with ZEN 2 (blue edition) software of Carl Zeiss LSM800 confocal laser scanning microscopy.
Data analysis	NMR spectra were analyzed using MestreNova LITE v14.0.0.0-23239 software (Mestre lab Research S.L.) and JEOL Delta v6.1.0 software. All the simulations were performed by using the Gaussian 16_A01 program package, and the HOMO and LUMO plots were visualized using VMD 1.9.3 software. Flow cytometry results were analyzed by FlowJo v10.0.7. Data analysis of imaging was done using viewMSOT 3.8 and the Living Image 4.3 software. Statistical calculations and data analysis were performed using OriginPro 2018 (64 bit) SRl b9.5.l.195 and GraphPad Prism 9.5 software.

For manuscripts utilizing custom algorithms or software that are central to the research but not yet described in published literature, software must be made available to editors and reviewers. We strongly encourage code deposition in a community repository (e.g. GitHub). See the Nature Portfolio guidelines for submitting code & software for further information.

Data

Policy information about availability of data

All manuscripts must include a data availability statement. This statement should provide the following information, where applicable:

- Accession codes, unique identifiers, or web links for publicly available datasets
- A description of any restrictions on data availability
- For clinical datasets or third party data, please ensure that the statement adheres to our policy

The authors declare that all the data supporting the findings of this study are available within the article and its Supplementary Information, and the full image dataset is available from the corresponding author upon request. Source data are provided with this paper. A reporting summary for this article is also available as a Supplementary Information file.

Human research participants

Policy information about studies involving human research participants and Sex and Gender in Research.

Reporting on sex and gender	N/A
Population characteristics	N/A
Recruitment	N/A
Ethics oversight	N/A

Note that full information on the approval of the study protocol must also be provided in the manuscript.

Field-specific reporting

Please select the one below that is the best fit for your research. If you are not sure, read the appropriate sections before making your selection.

☒ Life sciences ☐ Behavioural & social sciences ☐ Ecological, evolutionary & environmental sciences

For a reference copy of the document with all sections, see nature.com/documents/nr-reporting-summary-flat.pdf

Life sciences study design

All studies must disclose on these points even when the disclosure is negative.

Sample size	Animal experiments were performed in compliance with the regulations of Ethics Committee of Laboratory Animal Center of South China Agricultural University (Approval No. 2020d076) and the Guidelines for Care and Use of Laboratory Animals of the Institutional Animal Care and Use Committee of Nanyang Technological University (NTU-IACUC) (Approval No. A19016). No effect size was predetermined, but sample sizes ($n \geq 3$) were chosen based on previous experience with the animal models or following convention of the methods. We used G*power analysis to calculate and ensure the sample sizes fulfill adequate power ($p > 0.8$). According to the experimental data and sample size (n), P value and effect size were calculated and the power was then calculated. If it is higher than 80%, demonstrating the sample size is adequate.
Data exclusions	No data was excluded from this study.
Replication	Experiments were repeated at least three independent experiments with similar results. All experiments were reproduced to reliably support conclusions stated in the manuscript.
Randomization	Allocation was random.
Blinding	The investigators were blinded to group allocation during data collection and analysis.

Reporting for specific materials, systems and methods

We require information from authors about some types of materials, experimental systems and methods used in many studies. Here, indicate whether each material, system or method listed is relevant to your study. If you are not sure if a list item applies to your research, read the appropriate section before selecting a response.

Materials & experimental systems

n/a	Involved in the study
☐	☒ Antibodies
☐	☒ Eukaryotic cell lines
☒	☐ Palaeontology and archaeology
☐	☒ Animals and other organisms
☒	☐ Clinical data
☒	☐ Dual use research of concern

Methods

n/a	Involved in the study
☒	☐ ChIP-seq
☐	☒ Flow cytometry
☒	☐ MRI-based neuroimaging

Antibodies

Antibodies used	Rabbit anti-CD44 (cat no. A16807) and Rabbit anti-MMP-9 (cat no. A11147) were purchased from ABclonal Inc. Rabbit anti-MMP-2 (cat no. ET1606-4) and anti-IL-6 [cat no. EM1701-45] were purchased from HuaBio. Rabbit anti-CD206 (cat no. ab300621) and Alexa Fluor 488-labeled Goat Anti-Rabbit IgG H&L [catalog NO.: ab150077] were purchased from Abcam Inc. Mouse anti-β actin (cat no. BS6007M), goat anti-rabbit IgG(H+L) HRP (cat no. BS13278) and goat anti-mouse IgG H+L HRP (cat no. BS12478) were purchased from Bioworld.
Validation	All antibodies were used in the study according to the profile of manufacturers. Antibody validation was validated by the supplier and confirmed in Figure 5n, 6h, 6i, 7k, 7l, 7m and Supplementary Figures 53. Specifically, we relied on the references listed on the manufacture's website:<br>1. https://abclonal.com/catalog-antibodies/CD44RabbitpAb/A16807<br>2. https://abclonal.com/catalog-antibodies/MMP9RabbitpAb/A11147<br>3. https://www.huabio.com/products/mmp2-antibody-clone-si15-04-recombinant-monoclonal-et1606-4<br>4. https://www.huabio.com/products/il6-antibody-clone-1-6-monoclonal-em1701-45<br>5. https://www.abcam.com/products/primary-antibodies/mannose-receptor-antibody-epr25215-277-ab300621.html<br>6. https://www.abcam.com/products/secondary-antibodies/goat-rabbit-igg-hl-alexa-fluor-488-ab150077.html<br>7. https://www.citeab.com/antibodies/2209947-bs6007m-actin-4d3-monoclonal-antibodyn-a<br>8. https://www.citeab.com/antibodies/8909820-bs13278-goat-anti-rabbit-igg-h-l-hrp<br>9. https://www.citeab.com/antibodies/8909801-bs12478-goat-anti-mouse-igg-h-l-hrp

Eukaryotic cell lines

Policy information about cell lines and Sex and Gender in Research

Cell line source(s)	Human embryonic kidney normal cells (HEK 293), Mouse fibroblast cell line (L929), human breast adenocarcinoma epithelial cells (MDA-MB-231), human bladder cancer cell line (T24) and mouse mammary adenocarcinoma cell line (4T1) were purchased from ATCC. Human fetal hepatocyte line (L-02) was purchased from Ubigene.
Authentication	The suppliers routinely authenticate the cell lines by short tandem repeat profiling though the cell lines were not authenticated by our laboratory.
Mycoplasma contamination	Mycoplasma test was negative by the supplier using PCR-based assay.
Commonly misidentified lines (See ICLAC register)	No commonly misidentified cell lines were used.

Animals and other research organisms

Policy information about studies involving animals; ARRIVE guidelines recommended for reporting animal research, and Sex and Gender in Research

Laboratory animals	The male and female Balb/c mice (5-6 weeks) were purchased from Guangdong Medical Laboratory Animal Center (China) and InVivos Pte. Ltd. (Singapore). The animals were housed in sterile cages within laminar airflow hoods at 24 °C, 45-65% humidity in a specific pathogen-free room with a 12 h light/12 h dark schedule and fed autoclaved chow and water ad libitum.
Wild animals	No wild animals were used in this study.
Reporting on sex	This study did not involve sex consideration. Male or female mice were used to establish animal models according to literatures in the field.
Field-collected samples	This study did not involve samples collected from field.
Ethics oversight	The in vivo experiments were approved and conducted in compliance with the regulations of Ethics Committee of Laboratory Animal Center of South China Agricultural University (Approval No. 2020d076) and the Guidelines for Care and Use of Laboratory Animals of the Institutional Animal Care and Use Committee of Nanyang Technological University (NTU-IACUC) (Approval No. A19016).

Note that full information on the approval of the study protocol must also be provided in the manuscript.

Flow Cytometry

Plots

Confirm that:

- [x] The axis labels state the marker and fluorochrome used (e.g. CD4-FITC).
- [x] The axis scales are clearly visible. Include numbers along axes only for bottom left plot of group (a 'group' is an analysis of identical markers).
- [ ] All plots are contour plots with outliers or pseudocolor plots.
- [ ] A numerical value for number of cells or percentage (with statistics) is provided.

Methodology

Sample preparation	Flow cytometry was just used to study the cellular uptake of the nanoagent DXBTZ-CB[8]/CSA. Briefly, MDA-MB-231 cancer cells were seeded onto six-well plates at 2 × 10^5 cells per milliliter and allowed to culture for 24 h before treatments. Except for cells from the control well (0 min), cells in the other five wells were exposed to DXBTZ-CB[8]/CSA nanoparticles (50 µg mL^-1) and incubated for 15, 30, 45, 60, and 90 min, respectively. Afterwards, the treated cells were washed, trypsinized and centrifuged. Then, the cells were resuspended in PBS, and approximate 10,000 cells were recorded and analyzed.
Instrument	Fortessa X20 (BD Biosciences)
Software	FlowJo software package (version 10.0.7)
Cell population abundance	No cell sorting was performed in this study.
Gating strategy	In general, Cells were first determined through FSC-A vs. SSC-A to remove debris and free nanoparticles. Single cells were further determined through FSC-A vs. FSC-H.

- [x] Tick this box to confirm that a figure exemplifying the gating strategy is provided in the Supplementary Information.
